# Climate model differences contribute deep uncertainty in future Antarctic ice loss

**DOI:** 10.1126/sciadv.add7082

**Published:** 2023-02-15

**Authors:** Dawei Li, Robert M. DeConto, David Pollard

**Affiliations:** ^1^ School of Oceanography, Shanghai Jiao Tong University, Shanghai 200030, China.; ^2^ Department of Geosciences, University of Massachusetts Amherst, Amherst, MA 01003, USA.; ^3^ MNR Key Laboratory for Polar Science, Polar Research Institute of China, Shanghai 200136, China.; ^4^ Shanghai Key Laboratory of Polar Life and Environment Sciences, Shanghai Jiao Tong University, Shanghai 200030, China.; ^5^ Earth and Environmental Systems Institute, Pennsylvania State University, University Park, PA 16802, USA.

## Abstract

Future projections of ice sheets in response to different climate scenarios and their associated contributions to sea level changes are subject to deep uncertainty due to ice sheet instability processes, hampering a proper risk assessment of sea level rise and enaction of mitigation/adaptation strategies. For a systematic evaluation of the uncertainty due to climate model fields used as input to the ice sheet models, we drive a three-dimensional model of the Antarctic Ice Sheet (AIS) with the output from 36 climate models to simulate past and future changes in the AIS. Simulations show that a few climate models result in partial collapse of the West AIS under modeled preindustrial climates, and the spread in future changes in the AIS’s volume is comparable to the structural uncertainty originating from differing ice sheet models. These results highlight the need for improved representations of physical processes important for polar climate in climate models.

## Teaser

Climate models make a contribution comparable to ice sheet models for the uncertainty in future Antarctic ice loss.

## INTRODUCTION

Fluctuations of global mean sea level (GMSL) over the past few million years have been dominated by glacial-interglacial cycles. During the Last Glacial Maximum (21 ka ago), formation of the Laurentide Ice Sheet in North America and the Fennoscandian Ice Sheet in Northern Europe and, to a lesser extent, the expansion of Greenland and Antarctic ice sheets contributed to a ∼120-m drop in GMSL relative to today (*1*). GMSL during the interglacials was comparable to, although sometimes higher than, the present-day sea level. In contrast, the Last Interglacial (LIG; 129 to 116 ka ago) was not much warmer than preindustrial (−0.4° to 1.3°C) (*2*), but GMSL was 6 to 9 m higher (*3*, *4*), of which ∼3.1 to 6.1 m may have been contributed by the Antarctic Ice Sheet (AIS) (*2*, *5*). While the LIG is not a precise analog of future sea level, as Earth’s orbital parameters and polar insolation forcing likely played an important role (*6*), it still hints at a worrisome potential for future sea level rise (SLR) given the ∼1.2°C warming that has already occurred.

At a rate of 3.58 mm year^−1^ over the period 2006–2015, the rise in GMSL is accelerating and is now dominated by melting of land ice, including glaciers and ice sheets (*7*). Projections of SLR over the 21st century and beyond have been made for various emission scenarios, but they are subject to substantial uncertainty, which becomes greater in scenarios with higher greenhouse gas emissions and hence more warming. Under the assessment by the Sixth Assessment Report of the Intergovernmental Panel on Climate Change (IPCC-AR6), following the high greenhouse gas emission scenario of Shared Socioeconomic Pathway 5-8.5 (SSP5-8.5) (*8*), SLR is likely to reach 0.63 to 1.01 m by 2100, of which 0.03 to 0.34 m is expected to be contributed by the AIS (*9*). In contrast, another recent statistical analysis of multimodel ice sheet simulations indicates a smaller future contribution from Antarctica (−0.01 to 0.1 m likely range) under similar SSP5-8.5 forcing (*10*), highlighting ongoing uncertainty.

As the largest source of uncertainty of SLR beyond 2100, ice loss from the AIS has evaded robust projections. Much of this uncertainty can be attributed to the diversity of numerical ice sheet models (ISMs), which differ not only in spatial resolution, equations of stress balance, numerical schemes, and initialization methods but also in their treatment of key physical processes including grounding line migration, calving, surface mass balance (SMB), and basal processes. The associated uncertainty in the AIS’s response to climate warming has been explored in a number of model intercomparisons, such as the Ice Sheet Model Intercomparison Project (ISMIP; the most recent phase being ISMIP6) (*11*–*16*). These projects have provided valuable insights by focusing on the difference in ISMs’ response to prescribed changes in climate boundary conditions. For instance, ISM initialization experiments show good agreement in the AIS’s response to changes in SMB, but a much greater spread in the response to ice shelf basal melt (*13*). Designed to assess how responses differ across the spectrum of ISMs under a nonexhaustive suite of modeled climates, ISMIP6 drove a variety of ISMs with climate fields from a subset of Coupled Model Intercomparison Project Phase 5 (CMIP5) models. As the succeeding generation CMIP6 model output became available to the climate research community, ISM intercomparison projects would benefit from using a more comprehensive set of climate models to take into account a wider and up-to-date range of intermodel uncertainty.

It has been recognized that structural differences between climate models can produce divergent quasi-equilibrium states for the AIS in experiments where ISMs are forced by the output of climate models (*17*); however, there has been no comprehensive assessment of the uncertainty in projected future states of the AIS using the latest generation climate models. In existing ISM intercomparison projects, decentralized model development gives rise to ISMs across a wide spectrum, while often a small subset of available climate models is included to provide climate boundary conditions. Here, we take a complementary approach to evaluate the uncertainty in projected change of the AIS and its contribution to GMSL by driving a single three-dimensional ISM (*18*) with climate fields from 36 climate models in the CMIP6 archive. The ISM is fine-tuned so that it closely simulates the observed state of the AIS and rates of ice loss under present-day climate conditions (Experiment OBS_INV) (*5*). Assuming that the ISM is a “perfect” representation of the real AIS, the spread in ISM output reflects the uncertainty associated with past and future climate changes simulated by these CMIP6 models. Such “perfect model” framework has been widely used in climate research to evaluate model predictability, the performance of bias correction and statistical downscaling, etc. (*19*, *20*)

A series of ISM experiments under this perfect model framework, as documented in Table 1, were carried out to assess the effect of biases in modeled climate on the AIS’s equilibrium state and the uncertainty in past and future trajectories of the AIS due to divergent climate sensitivities displayed by CMIP6 models. We also provide additional ISM experiments to discuss an ice sheet instability mechanism, the effect of observed multidecadal warming of Antarctic subsurface ocean on the AIS, and an alternative modeling strategy with the ISM calibrated per climate model.

**Table 1.** List of experiments. BSC, basal sliding coefficient; OMF, ocean melt factor; BC, bias-corrected; MICI, marine ice cliff instability.

*#*	Experiment (suite) name	Climate forcing	Init. cond.	Parameters (TPD/OMF/BSC)	Length (years)	Description/aim
0	OBS_INV	PD observation^*^	PD^†^	−1.0/5.0/vary	100,000	Adjusting ISM BSC to simulate an AIS close to present-day under observed climate.
1	OBS_CTL	PD observation	PD	−1.0/5.0/Exp 0	10,000	ISM control run under observed climate for validating the ISM’s performance.
2	CMIP6_RAW_PI_CTL	CMIP6 raw PI^‡^	PD	−1.0/5.0/Exp 0	10,000 (×36)	Assessing the effect of raw CMIP6 climate on simulated AIS equilibrium state.
3	CMIP6_BC_PI_CTL	CMIP6 BC PI	PD	−1.0/5.0/Exp 0	15,000 (×36)	Assessing the spread in simulated AIS due to differences in BC CMIP6 preindustrial climates.
4	CMIP6_BC_1850-2100	CMIP6 BC 1850–2100^§^	Exp 3	−1.0/5.0/Exp 0	250 (× 36)	Quantifying the spread in simulated AIS due to differences in BC CMIP6 1850–2100 climates.
5	CMIP6_BC_1850-2100_NO_MICI	CMIP6 BC 1850–2100	Exp 3	−1.0/5.0/Exp 0/MICI off	250 (×36)	Same as Exp 4 but with MICI-related processes turned off for evaluating the effect of MICI.
6	CMIP6_BC_MMM	CMIP6 BC MMM 1850–2100	Exp 3	−1.0/5.0/Exp 0	250	Same as Exp 4 but a single run forced by CMIP6 multimodel mean climate.
7	CMIP6_BC_MMM+OBS	CMIP6 BC MMM 1850–2100, 1980–2019 OBS	Exp 3	−1.0/5.0/Exp 0	250	Evaluating the effects of multidecadal trends in observed Antarctic climate on the AIS.
8	CMIP6_RAW_PD_TPD	CMIP6 raw PD	PD	Vary/5.0/Exp 0	1 (× 36)	Tuning TPD for each CMIP6 model to get the observed rates of surface melt under modeled PD climate.
9	CMIP6_RAW_PD_OMF	CMIP6 raw PD	PD	0.0/vary/Exp 0	1 (×36)	Tuning OMF for each CMIP6 model to get the observed sub-ice melt under modeled PD climate.
10	CMIP6_RAW_PI_INV	CMIP6 raw PI	PD	Exp 8/Exp 9/vary	80,000 (×36)	Tuning BSC for each CMIP6 model to simulate a realistic AIS under preindustrial climate.
11	CMIP6_RAW_PI_CTL2	CMIP6 raw PI	PD	Exp 8/Exp 9/Exp 10	250 (× 36)	Quantifying drifts in the AIS under CMIP6 preindustrial climates.
12	CMIP6_RAW_1850-2100	CMIP6 raw 1850–2100	PD	Exp 8/Exp 9/Exp 10	250 (×36)	Same as Exp 4 but forced with raw CMIP6 climate and the ISM tuned separately for each CMIP6 model.

*PD observation: Present-day (PD) climatological mean (averaged over 1981–2010), including monthly surface air temperature and precipitation from ERA5 and annual 400-m ocean temperature from World Ocean Atlas 2018 (WOA18).

†PD: PD observed state of the AIS specified in Bedmap2.

‡PI: Preindustrial climate. However, because not all CMIP6 models used in this study have a PI control run, climate fields averaged over the period 1850–1869 of historical runs are used here as approximately preindustrial.

§1850–2100: Historical (1850–2014) and SSP5-8.5 (2015–2100) scenarios are combined to provide climate forcings for each year over the period 1850–2100.

## RESULTS

### Near-equilibrium AIS under raw CMIP6 climates

In the experiment set CMIP6_RAW_PI_CTL (Table 1 and Methods), we investigate the effect of differing modeled preindustrial climates on the equilibrium state of the AIS. The AIS is initialized from the end state of an inverse simulation (*21*), which infers the characteristics of the AIS bed required to simulate a realistic AIS under the present-day observational climate (Experiment OBS_INV). Here, “present day” refers to the 3-year period 1981–2010, within the historical period per the CMIP6 protocol (1850–2014); “preindustrial” is defined as the 20-year period 1850–1869, under the assumption that, within the first 20 years, anthropogenic forcings had not changed the climate substantially from its pre-1850 state.

**Fig. 1.**
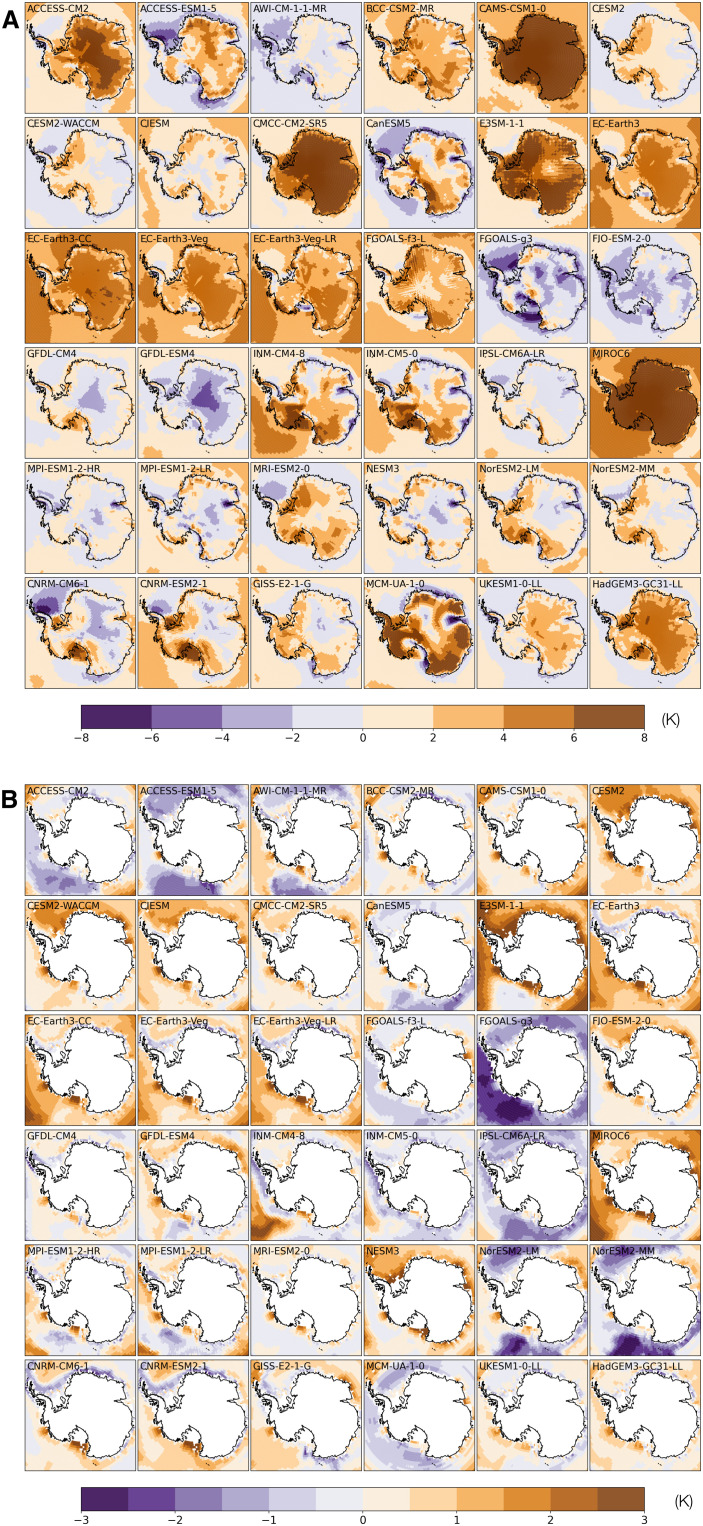
Difference between modeled climate fields and observations. (**A**) Difference in January surface air temperature between 36 CMIP6 climate models and observations (ERA5). (**B**) Difference in annual mean 400-m ocean temperature between 36 CMIP6 climate models and observations [World Ocean Atlas 2018 (WOA18)]. Climate fields from CMIP6 models and observations are averaged over the period 1981–2010.

A comparison of simulated present-day summer [December to February (DJF)] near-surface air temperature (*T*_2m_) and 400-m annual ocean potential temperature (θ_400m_) reveals substantial differences between CMIP6 models (Fig. 1). Deviations of modeled temperatures from observation display distinct spatial heterogeneities. For instance, ACCESS-ESM-1-5 has a warm bias in DJF *T*_2m_ over the ice sheet but a cold bias over the ocean relative to ERA5 (Fig. 1A). Modeled θ_400m_ can be too warm in one ocean sector but too cold in others (Fig. 1B). In addition to subsurface ocean temperatures, air temperature also strongly affects the stability of ice shelves in summer, when most surface melt occurs under the present-day climate. Rheological properties of the glacial ice are, in contrast, affected mainly by annual mean temperature, because seasonal variations in temperature only penetrate ∼1 m into the ice, a tiny fraction of the typical thickness of the ice sheet or ice shelves. We find substantial intermodel variation in simulated *T*_2m_ with a warm bias over the ice shelves as much as 8°C in some models (Fig. 1A).

The difference between simulated annual precipitation and that from ERA5 reanalysis dataset (*22*) generally shows patterns consistent with surface air temperature biases, with warmer models experiencing greater precipitation and vice versa (see the Supplementary Materials). The difference between modeled and observed subsurface ocean temperature at 400 m is less notable, but it is still substantial, as ice shelf basal melt rates are sensitive to ocean temperatures. Under the parameterization scheme used in the ISM, the basal melt rate has a quadratic dependence on θ_400m_ (see Methods) so even modest intermodel differences can substantially change the basal mass balance of ice shelves with important consequences for the buttressed ice upstream.

**Fig. 2.**
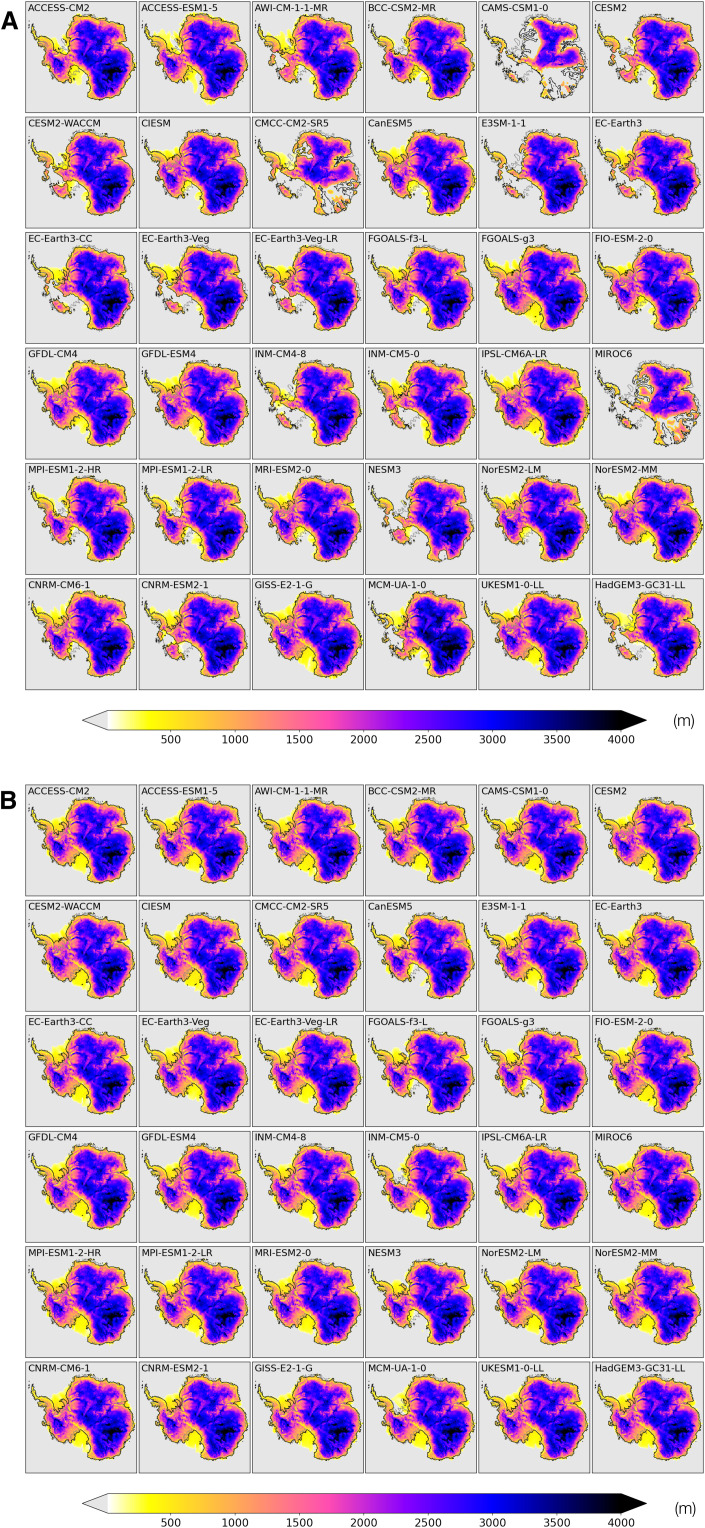
Simulated ice sheets under CMIP6 preindustrial climates. (**A**) Ice thickness by the end of the control runs forced by raw preindustrial climates from 36 CMIP6 models (Experiment CMIP6_RAW_PI_CTL). (**B**) Same as (A) but for simulations forced by bias-corrected CMIP6 preindustrial climates (Experiment CMIP6_BC_PI_CTL).

**Fig. 3.**
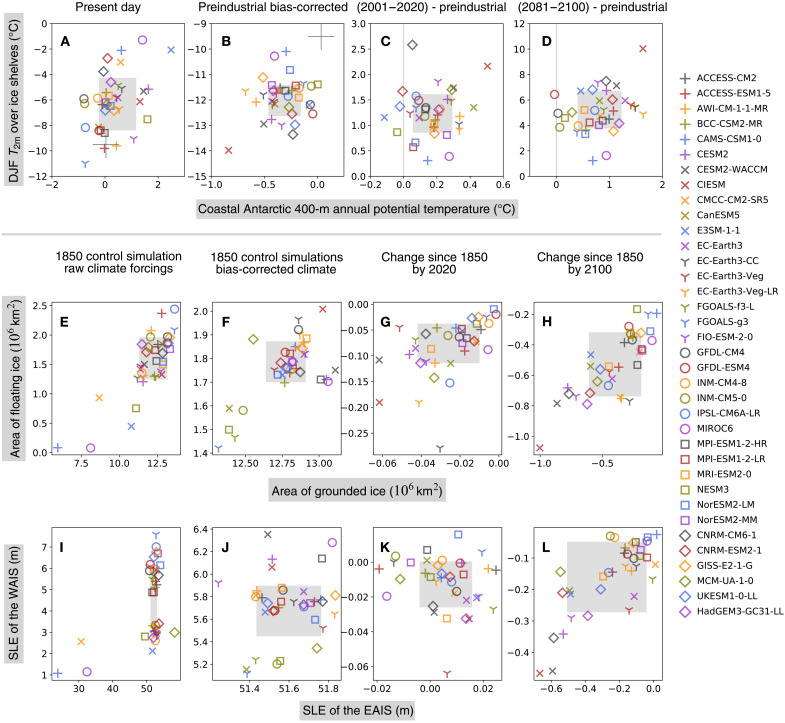
Intermodel differences in CMIP6 climates and simulated AIS. Scatter plots show intermodel differences in modeled Antarctic climate and resulting states of the AIS forced by 36 CMIP6 climate models, represented by markers of different shapes and colors. (**A** to **D**) DJF near-surface (2-m) air temperature (*T*_2m_) (°C, vertical axes) averaged over ice shelves against Antarctic coastal ocean potential temperature at 400-m (°C, horizontal axes), (**E** to **H**) area of floating ice (10 × 10^6^ km^2^, vertical axes) against area of grounded ice (10^6^ km^2^, horizontal axes), and (**I** to **L**) contributions to GMSL change from the West AIS (WAIS) (m, vertical axes) against the East AIS (EAIS) (m, horizontal axes). (A) and (B) shows the raw (uncorrected) and bias-corrected preindustrial climates, respectively; (C) and (D) show the changes relative to the 1850–1869 period by year 2020 and 2100. Similarly, (E) and (F) and (I) and (J) show ISM results forced by the raw and bias-corrected climates, respectively; (G) and (H) and (K) and (L) show ice sheet changes from the initial preindustrial state at 2020 and 2100, respectively, forced by bias-corrected climates (Experiment CMIP6_BC_1850-2100). Gray squares show 16 to 84 percentile range of intermodel spread.

A myriad of quasi-equilibrium states of the AIS are reached in 10,000-year runs forced by 36 CMIP6 models’ preindustrial climates (Fig. 2A). In 17 simulations, near-complete collapse of the West AIS (WAIS) contributes >3 m of the GMSL rise (Fig. 3I). In addition, climate forcing from three models with a strong warm bias produces substantial retreat of the East AIS (EAIS), contributing >15 m of the GMSL rise. Climate models with a cold bias in subsurface temperature θ_400m_, in contrast, generally drive the ISM toward a quasi-equilibrium state with an expanded ice sheet and seaward advance of grounding lines onto continental shelves (Figs. 2A and 3).

These ISM control experiments highlight the room for improvement in CMIP6 models’ performance in the Antarctic region. The simulations also corroborate the established wisdom that the WAIS is especially sensitive to ocean temperatures: For example, the climate model NESM3 has a mean circum-Antarctic warm bias of 1.5°C in θ_400m_ (Fig. 3A), but this is sufficient to drive a partial collapse of the WAIS in the ISM on long time scales (Fig. 3I).

Despite that unrealistic AIS geometries were simulated by under many CMIP6-modeled climates, these experiments are not designed for evaluating CMIP6 climate models’ performance over Antarctica. Here, we have regarded reanalysis datasets as the “observational truth,” serving as a reference climate for calibrating the ISM model parameters, including the ocean melt rate coefficient and the basal sliding coefficients (BSCs). Because of the scarcity of observations available for Antarctica, reanalysis datasets may have substantial departures from the true climate state in some regions. These control experiments are run for 10,000 years, allowing the AIS to reach a quasi-equilibrium, but it is not clear how close the AIS was to such a state before the dawn of the Industrial Revolution, when anthropogenic climate forcing started to emerge. Furthermore, Earth’s orbital parameters drift substantially over 10,000 years, and the AIS is expected to respond accordingly. The availability of CMIP6 historical simulations, dating back to only 1850, makes the quasi-equilibrium assumption necessary for conducting an intercomparison of the AIS forced by different climate models, but the intrinsic uncertainty in the AIS’s natural variability cautions against judging these climate models based on their respective ISM simulations.

### Near-equilibrium AIS under bias-corrected CMIP6 climates

The diverse polar climates simulated by CMIP6 models render the above approach unsuitable for assessing the uncertainty in the AIS’s future trajectory. An alternative strategy is to bias-correct CMIP6 climates against present-day observations. Spatially varying biases in CMIP6 monthly climate fields are calculated and subtracted from the raw model output (Methods). In this approach, we essentially remove CMIP6 models’ biases in present-day climates and focus on their changes from the reference period. However, because of CMIP6 models’ differing sensitivities to anthropogenic forcings, bias-corrected preindustrial climates for Antarctica still display significant intermodel variations, showing a large intermodel spread in preindustrial *T*_2m_ and θ_400m_. Mean DJF *T*_2m_ over ice shelves is up to 4.5 K lower than the present-day reference period. Simulated preindustrial annual mean θ_400m_ averaged along the Antarctic coast is up to 1 K lower than present-day (Fig. 3B). Note that warming proceeds at a faster pace in the atmosphere than the subsurface ocean, underscoring complex processes at play in the Southern Ocean, where vigorous convection and upwelling around Antarctica may suppress the pace of warming (*23*).

In the experiment set CMIP6_BC_PI_CTL, the ISM is initiated from the present-day AIS and runs for 15,000 years until it reaches a quasi-equilibrium but forced with CMIP6 bias-corrected preindustrial climates. Compared with the initial state, the modeled preindustrial AIS in quasi-equilibrium generally shows thinning of the EAIS, consistent with reduced snowfall in a colder preindustrial climate. Under most CMIP6 models, ice shelves around the AIS expand, which is also consistent with lower preindustrial ocean temperatures. Intermodel differences in ice volume of the EAIS and the WAIS are 0.6- and 1.2-m sea level equivalent (SLE), respectively (Fig. 3I).

### Projected changes in Antarctic climate and the AIS

**Fig. 4.**
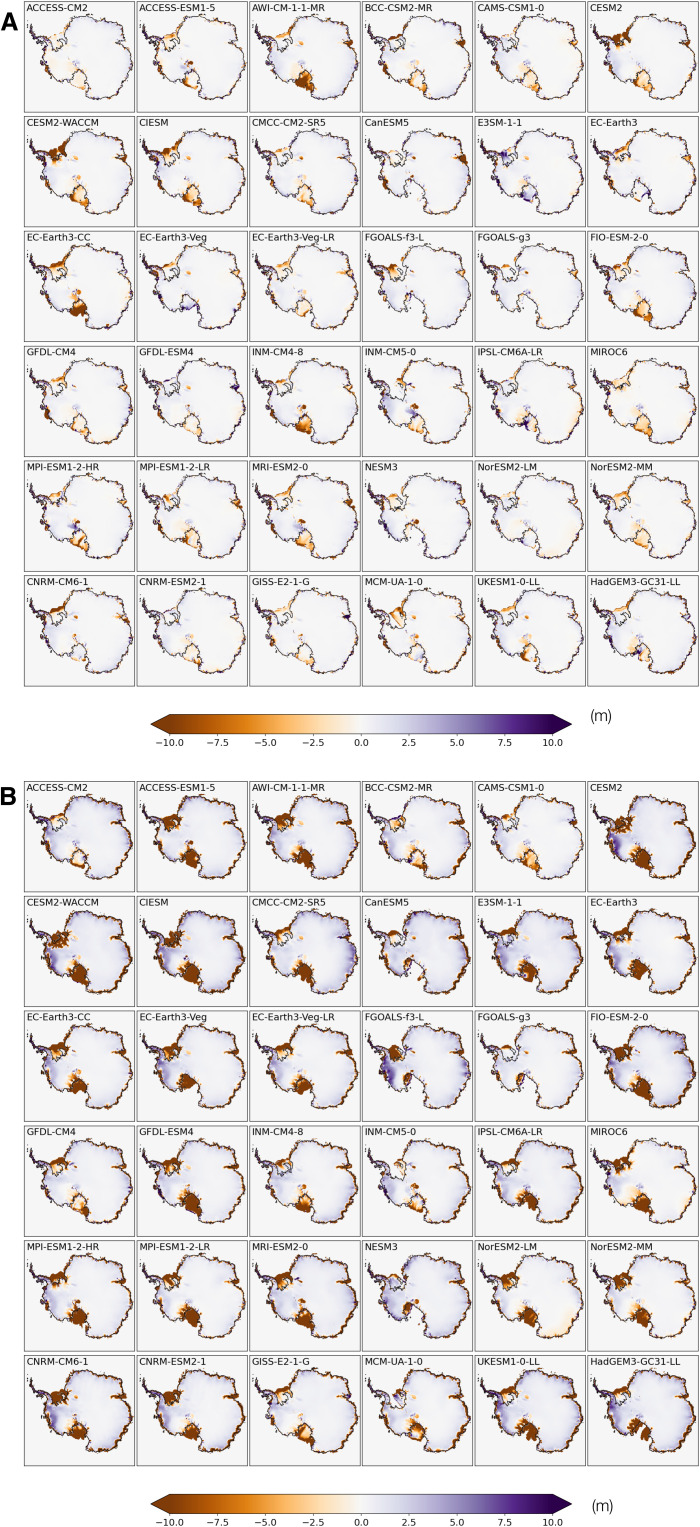
Simulated changes in ice thickness since 1850. (**A**) Changes in ice thickness since 1850 by year 2020 in simulations transiently forced by bias-corrected historical + SSP5-8.5 climates from 36 CMIP6 models (Experiment CMIP6_BC_1850–2100). (**B**) Same as (A) but for year 2100.

Under the SSP5-8.5 scenario, all CMIP6 models included in this study show substantial warming relative to preindustrial in both *T*_2m_ and θ_400m_ over this century (Figs. 3D and 4). DJF *T*_2m_ averaged over all Antarctic ice shelf surfaces increases by 0.3 to 2.6 K in 2020 and by 1 to 10 K in 2100; θ_400m_ averaged along the Antarctic coast increases by −0.1 to 0.5 K in 2020 and up to 1.6 K in 2100 (Fig. 3, C and D). The amplitude of warming in climate models reveals dependence on the state of simulated reference climate. For instance, CAMS-CSM1-0 and MIROC6 are among the models with the greatest warm bias in *T*_2m_ (Figs. 1A and 3A), but they also show the least warming (<2 K) by 2100. One of the contributing factors might be that, in preindustrial climates, these models are mostly free of austral summer sea ice, reducing the strength of sea ice-albedo feedback in future warming scenarios.

Experiment set CMIP6_BC_1850-2100 are 250-year ISM runs under transient bias-corrected CMIP6 climates in combined historical (1850–2014) and SSP5-8.5 (2015–2100) scenarios, with the ice sheet initiated from the respective 15,000-year control simulation under the bias-corrected preindustrial climate described previously (Experiment CMIP6_BC_PI_CTL). Climate fields are bias-corrected and drive the ISM year by year, so that an evolution of the AIS is obtained for each CMIP6 model. In this approach, we essentially remove each CMIP6 model’s bias in simulated present-day climate and focus on the course of simulated climate change and associated impact on the AIS, especially on the uncertainty in the AIS’s future projections.

**Fig. 5.**
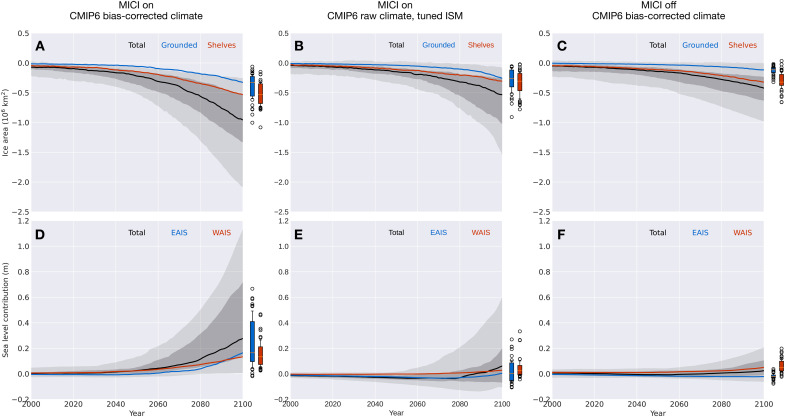
Simulated changes in the AIS’s area and sea level contribution. Top panels show changes in ice area relative to the preindustrial, where black, blue, and red lines represent all, grounded, and floating (shelf) ice, respectively. Bottom panels show changes in the AIS’s contribution to GMSL rise, where black, blue, and red lines are for the whole AIS, the EAIS, and the WAIS, respectively. Results from experiments with marine ice cliff instability (MICI) processes and forced with bias-corrected CMIP6 model climate (Experiment CMIP6_BC_1850-2100) are shown in the left column. Middle column shows results from experiments with MICI processes but forced with raw CMIP6 model climate, while the ISM is tuned separately for each CMIP6 model (Experiment CMIP6_RAW_1850-2100) (see Methods and figs. S13 to S16). Right column is for experiments forced with bias-corrected CMIP6 model climate, while MICI-related processes are turned off (Experiment CMIP6_BC_1850-2100_NO_MICI). In each panel, the full spread (0 to 100th percentile) in 36 simulations is shaded in light gray, and 16th to 84th percentile are in darker gray. The full spread and 16th to 84th percentile of respective variables for grounded ice/EAIS and floating ice/WAIS at 2100 are shown as blue and red boxplots, respectively, to the right of each panel.

Projected changes in the Antarctic climate from all CMIP6 models drive a reduction in both AIS volume and the extent of ice shelves (Figs. 3 and 5). The magnitude of ice loss, however, shows a large intermodel spread. CIESM shows the largest warming in atmospheric and oceanic temperatures and drives the most intense Antarctic ice loss. CESM2, CESM2-WACCM, and CNRM-CM6-1 are among the models with the largest warming in *T*_2m_ by 2100 (Fig. 3C); they also drive some of the largest reductions in ice volume. Counterintuitively, the four variants of EC-Earth3 show greater oceanic warming, but they produce much less 21st century ice loss (Fig. 3, D and H). In the previous three models, ice surface melting and the loss of ice shelves overshadow sub-ice melting due to oceanic warming, which has been the focus of most recent studies on the sensitivity of the AIS, especially its marine-based WAIS portion (*24*). Climate models with the strongest atmospheric warming also produce the largest WAIS retreat, raising the GMSL by >0.25 m by 2100 (Fig. 3L). A contributing factor for this emerging correlation may be that the ISM used in this study resolves hydrofracturing and ice cliff failure processes, which make the ice shelves prone to collapse triggered by surface melting and thus increase the ISM’s sensitivity to atmospheric warming.

These 250-year AIS simulations using bias-corrected climates from 36 CMIP6 models reveal both accelerating retreat of the AIS and increasing uncertainty in its future trajectory. Relative to its preindustrial state, the multimodel median rate of ice loss increases by almost an order of magnitude from 2020 to 2100 (Fig. 3, G and H). EAIS and WAIS display contrasting changes over the early stage of warming before 2020: The WAIS loses mass and contributes to a SLR under all CMIP6 models’ bias-corrected climate trajectories, while, under most CMIP6 models (27 of 36), the EAIS gains mass and draws down GMSL (Fig. 3K). Between 1850 and 2020, the EAIS produces a small negative (~−0.01 m) multimodel median contribution to GMSL rise, while reduction in the WAIS is more consistent across models. As the 21st century warming proceeds, the EAIS is expected to reverse its trend later and begin to lose mass (Fig. 5C). By 2100, the multimodel median reduction in ice area increases to 6 × 10^5^ km^2^, and the multimodel median sea level contribution of the AIS approaches 0.3 m (Fig. 5), with the highest modeled SLR exceeding 1 m. The full range of the AIS’s sea level contribution by 2100 greatly exceeds its multimodel median value as a result of the strong nonlinearity in the ice sheet’s response to temperature change. While the CMIP multimodel mean/median has been shown to produce an accurate representation of modern climate state, and multimodel median sea level projections remain more policy relevant than end-members, we should beware of the existence of low-probability, high-consequence scenarios in future SLR.

## DISCUSSION

### Effect of MICI on projected ice loss

The projected MMM rise in GMSL contributed by the AIS and associated uncertainty in these CMIP6-driven ISM simulations is noticeably greater than those assessed by ISMIP6 (*15*) and IPCC-AR6 (*9*). A possible factor might be the “marine ice cliff instability” (MICI) mechanism, which is accounted for in our ISM but has not been widely implemented in other ISMs. The ISM used in this study includes optional hydrofracturing and ice cliff failure mechanisms (*25*), which may give rise to MICI (*5*, *26*, *27*) under strong future warming scenarios but not in preindustrial and present-day climate conditions. MICI is a newly proposed mechanism, and there have been ongoing discussions concerning its validity. Self-sustaining ice loss triggered by MICI has been proposed to be necessary for explaining the Antarctic contribution to sea level high stands during the LIG and the Pliocene (*5*, *28*) as well as the ice berg keel marks formed in deep water during the last deglaciation in the Amundsen Sea Embayment (*29*). On the other hand, some suggest that MICI is not well constrained and is not required to explain past sea level high stands (*30*), it may be mitigated by slow removal of ice shelves (*31*), and the progress of instability may be slowed by ice-mélange buttressing. Recent advances in modeling ice cliff failure reveal that MICI remains a feasible mechanism, but glacier models have shown a higher degree of complexity (*32*, *33*) compared to the parameterization scheme originally implemented in our ISM.

Although key parameters for hydrofracturing and cliff failure have been updated and constrained by sea level proxy data and observational records (*5*), considering their associated uncertainty, we also carried out alternative experiments without MICI processes (Exp. CMIP6_BC_1850-2100_NO_MICI). Without MICI, the ISM runs show smaller sea level contributions from the AIS by 2100, ranging from −0.05 to 0.2 m, with a median of 0.02 m, more in line with the findings of a recent study using statistical emulators of ISMs (*10*). In the absence of hydrofracturing and ice cliff failure, the warming in near-surface air temperature increases surface melt but does not trigger widespread collapse of ice shelves, and any tall ice cliffs that do emerge where ice shelves are lost remain intact in the model. Ignoring hydrofracturing and ice cliff failure processes puts our model in the lower range among ISMs in terms of its sensitivity to climate warming, so in these simulations without MICI, the resulting uncertainty in future sea level change reflects the combination of widely differing CMIP6 climate fields and a low-sensitivity ISM. However, even without MICI-related processes, the full range of climate-driven sea level uncertainty contributed by the AIS still amounts to 0.25 m by 2100, exceeding uncertainties from other major contributors, including sea water thermal expansion, mountain glaciers, and the Greenland Ice Sheet (*7*).

### Implications on observed ice sheet changes in recent decades

**Fig. 6.**
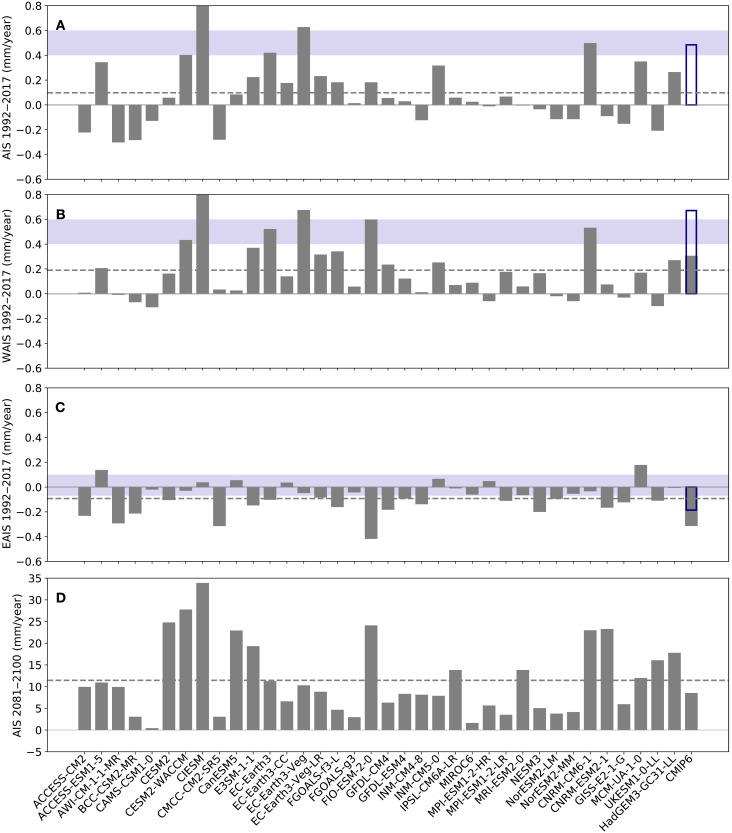
Simulated rates of GMSL change. (**A**) Contributions to the mean rate of change in GMSL during the IMBIE period (1992–2017) by the AIS in simulations forced by bias-corrected CMIP6 model climates, where the likely ranges estimated by IMBIE are marked by horizontal blue bars. (**B**) Same as (A) but for the WAIS. (**C**) Same as (A) but for the EAIS. (**D**) Same as (A) but for the late-century period 2081–2100 under the SSP5-8.5 scenario. Results from each model are shown in gray bars, and the horizontal dashed lines represent the multimodel mean. Gray bars labeled as “CMIP6” represent an ice sheet simulation forced by the multimodel mean climate fields of 36 models, while hollow blue bars are for a similar simulation but with forcings during 1980–2019 replaced by observed fields from ERA5 and WOA18.

Centennial and millennial trends in the AIS are dictated by long-term climate change, natural or anthropogenic, but internal variabilities of the climate system may still be important for multidecadal ice sheet changes, e.g., changes in polar ice sheets observed during the satellite era. Few of the ISM simulations driven by bias-corrected CMIP6 climates (Exp. CMIP6_BC_1850-2100) show an Antarctic contribution to GMSL over 1992–2017 consistent with that estimated by the Ice Sheet Mass Balance Intercomparison Exercise (IMBIE) team (*34*, *35*). Forced by the multimodel mean outputs from 36 CMIP6 models, net contribution by the AIS during the IMBIE period 1992–2017 is minimal (Fig. 6A).

In another simulation with the same climate forcing, but its 1980–2019 segment replaced by observational data (Exp. CMIP6_BC_MMM+OBS), Antarctica’s contribution to GMSL rise is more consistent with the IMBIE assessment, as a result of faster retreat of the WAIS and slower growth of the EAIS. A multidecadal warming trend since the 1970s in the circumpolar deep water (CDW) (fig. S12) (*36*), a relatively warm water mass circulating around Antarctica, may have enhanced basal melting of West Antarctic ice shelves. The ISM presents rates of ice loss comparable to IMBIE estimates when driven by the observed transient climate (fig. S12). Multimodel mean climate fields are essentially devoid of internal climate variabilities—provided that the number of models is large enough—due to cancellation of random phases from models. The observed multidecadal CDW warming trend, which may be partially caused by internal climate variability, cannot—and should not—be expected to be robustly reproduced in CMIP6 historical simulations, and its absence could be a factor for the generally small 1992–2017 trends from ISM simulations forced by CMIP6 models.

### ISM intercomparison projects

A number of modeling studies concerning the uncertainty in future SLR contributed by the AIS have been carried out. The ISMIP6-Antarctica project (*15*) used ISMs from 13 modeling groups and six CMIP5 climate models. A smaller subset of CMIP6 models, all with an equilibrium climate sensitivity (ECS) near the upper end of climate models, has been used in similar ways to assess the future GMSL contributions by ice sheets under different emission scenarios (*16*). The work presented here complements the scope of existing ISM intercomparison projects. We have included 36 climate models from the CMIP6 ensemble, which encompass a wider range of ECS and more fully represent the contemporary understanding of the climate system and its future changes. CMIP6 models are known to have an overall higher ECS compared with CMIP5 models, primarily as a result of stronger positive cloud feedbacks from refined cloud schemes (*37*). Although only one ISM is used in this study, we have provided contrasting simulations with and without MICI processes, differing substantially in the sensitivity to atmospheric warming.

Under the Representative Concentration Pathway (RCP) 8.5 scenario, a radiative forcing scenario similar to its CMIP6 successor SSP5-8.5, ISMIP6 simulations with 13 different ISMs give an Antarctic contribution to GMSL during the period 2015–2100 between −7.8 and 30 cm (*15*). In those simulations, WAIS retreat shows great variance among projections, up to 18-cm SLE, while the EAIS mass change varies between −6.1- and 8.3-cm SLE. These ISMIP6 projections present less ice loss and associated uncertainty compared with those in our simulations with the MICI mechanism, which is not considered in ISMIP6. Another contributing factor is the higher ECS of CMIP6 models used here, which generally warm more rapidly under SSP5-8.5 compared with CMIP5 models under RCP8.5.

### Effects of ISM calibration per climate model

Results discussed so far are all from ISM runs in a “single-ISM” framework, where the ISM is calibrated on the basis of observational data, with its parameters fixed for all CMIP6 climate models. Nonetheless, calibrating an ISM’s parameters so that, under a prescribed climate, it could simulate that a target ice sheet state is a common practice in the ice sheet modeling community, in which ISM parameters may absorb part of the spread in climate boundary conditions. Ice sheet intercomparison projects, e.g., ISMIP6, were carried out in similar ways, in which ISMs from decentralized development were calibrated separately with their own targets. To assess the effect of ISM tuning on projected Antarctic ice loss, we carried out a series of experiments to tune key ISM parameters for the preindustrial climate simulated by each CMIP6 model (Methods). This essentially results in multiple ISMs, each tailored for the respective CMIP6 model. We then run future projections of the AIS with the raw climate output from CMIP6 models, rather than bias-corrected climate as we did previously.

With this “multi-ISM” approach, the spread in simulated AIS forced by raw CMIP6 1850–2100 climate is smaller than that in single-ISM runs (Fig. 5). In comparison with ISMIP6 results, however, the dispersion of simulated Antarctic ice loss by 2100 in multi-ISM runs is still larger than that documented by ISMIP6-Antarctica. This may well be contributed to the more comprehensive set of climate models used in our study and CMIP6 models generally showing a higher climate sensitivity to elevated greenhouse gas levels, despite that the SSP5-8.5 scenario (*8*) used by CMIP6 models has slightly lower rates of greenhouse gas emissions than RCP8.5—its CMIP5 counterpart used by ISMIP6.

Some CMIP6 models with a high climate sensitivity happen to display a warm bias in simulated present-day Antarctic climate. For instance, CESM2-WACCM drives one of the largest Antarctic ice loss by 2100 (∼1.05 m) in the single-ISM run, but the number reduces to only ∼0.25-m SLE in the multi-ISM run. Examining the tuned ocean melt factor (OMF) (fig. S13), we can see that, to compensate the warm bias in CESM-WACCM’s 400-m ocean temperature, the OMF has to be reduced to 0.72, much smaller than the OMF (5.0) used in single-ISM runs, which was calibrated on the basis of present-day observations. This, of course, greatly reduces the ISM’s sensitivity to oceanic warming. In the case of MIROC6, which has an exceptionally large warm bias in the surface air temperature, the temperature offset in the ISM's positive-degree-day scheme (TPD) has to be increased to 4.64 K so that the modeled rate of surface meltwater production is around 100 Gt year^−1^. In other words, for the ISM tuned for MIROC6, ice and snow only melt at temperatures higher than 4.64 K. This is clearly unphysical but not an unexpected outcome of the tuning process. As warm bias in simulated modern polar climate is more prevalent than cold bias among CMIP6 models (Fig. 1A), tuning specifically for each CMIP6 model would generally reduce the ISM’s sensitivity to climatic warming and narrow the spread in projected ice loss.

Then, we come to the question whether this multi-ISM approach, in comparison with the single-ISM way, is more appropriate in assessing the uncertainty in projected Antarctic ice loss associated with climate models. The multi-ISM way hides climate models’ biases under tailored ISM parameter settings but may resort to parameters that are unphysical or contradicting to observational evidence.

### Implications for Earth system model development

The large spread in modeled polar climate in the current generation CMIP6 models would make it highly challenging to conduct intercomparisons of “Earth system models” with embedded, active ice sheets. It is not uncommon for climate models from different modeling centers to share components, and the same ISM or its close variants may be incorporated in several Earth system models. For instance, the Parallel Ice Sheet Model (PISM) is used in NASA GISS and MPI-ESM models, and the Grenoble ice sheet and land ice (GRISLI) model is used in CNRM-CM and IPSL-CM6 (*11*). Our ISM simulations forced by raw CMIP6 climates have demonstrated that, even with the same ISM, structural differences between atmosphere-ocean models can result in widely varying equilibrium states of the AIS. It has been recognized that simulated paleo–ice sheet volume, such as that during the mid-Pliocene, is highly dependent on climate model–based forcings (*17*).

Results from our study highlight that biases in simulated polar climate from state-of-the-art climate models are large enough to drive the AIS to equilibrium states distinctly different from the present day, although the ISM simulates a realistic AIS with observational climate data. This poses a serious challenge to the practice of using paleo sea level to constrain the parameters of ice sheet processes, irrespective of the accuracy of ice volume and sea level reconstructions (*38*).

Since the advent of numerical general circulation models in the 1960s, climate models have followed an evolutionary path of increasing complexity with ever more components added for explicit simulation (*39*). Spanning a hierarchy of models (*40*), climate modeling has now entered the Earth system model phase, where the most sophisticated models have added biogeochemical cycles and land ice sheets to the atmosphere-land-ocean system. Integrated ice sheet components embedded within Earth system models allow consistent simulations of crucial processes for polar climate change, e.g., the ice-albedo feedback, ice-elevation feedbacks associated with an evolving ice sheet topography, and the climate feedbacks associated with ice sheet meltwater (*41*–*43*). Results from our study, however, warn of substantial ongoing uncertainty among Earth system models with interactive ice sheets for the evaluation of future SLR. While progress has been made in ice sheet modeling, the uncertainty in future changes of the AIS and associated impacts on GMSL have not been reduced to a level needed for straightforward decision-making, and more work is required. Current greenhouse gas emissions put the climate on track of a >3°C warming by 2100, and the time window is shrinking for reducing carbon emissions to avoid rapid and unstoppable SLR (*5*). For more robust sea level projections, improved understanding of processes important for polar climate, including cloud radiative forcing and deep ocean circulations and mixing, is urgently needed.

## METHODS

### Ice sheet model

In this study, we use PSUICE3D (*18*), a numerical ISM with a hybrid approach to the dynamical equations governing ice sheet and ice shelf flow, which are described by the shallow ice and shallow shelf approximations, respectively, and are combined heuristically by an imposed mass flux condition across the grounding line (*44*). These hybrid ice dynamics capture the migration of grounding lines and essential mechanisms of ice sheet–ice shelf dynamics, e.g., the marine ice sheet instability (MISI) for an inward-deepening ice sheet bed, while allowing the model to be run on coarse grids (20 km in this study) so that a large ensemble of simulations can be carried out economically on centennial and millennial time scales and on continental spatial scales. Bedrock deformation under the weight of the ice sheet is represented by a local relaxation toward isostatic equilibrium and elastic lithospheric flexure. No explicit basal hydrology is implemented in the model other than allowing basal sliding where the basal temperature reaches the melt point. BSCs of the bed are obtained using an inverse method, in which the model is driven by present-day observational climate and the sliding coefficient at each grid point is tuned iteratively until the local ice thickness equilibrates toward the present-day observed value (*21*).

Ice sheet SMB is calculated as snowfall minus surface melt, while sublimation at the ice surface is ignored. The fraction of precipitation falling as snow is determined by a parameterized formulation based on the corresponding monthly surface air temperature *T*_a_ (*45*). Ice surface melt is calculated from *T*_a_ using a standard positive-degree-day (PDD) scheme with a coefficient of 0.005 m per degree-day, but the temperature baseline for zero melt (parameter TPD) is set at −1°C in single-ISM runs so that, under present-day climate, the total surface melt rate of Antarctic ice shelves is within the observational range (*46*).

Heavily parameterized in the current generation ISMs, ocean-induced ice shelf basal melt is recognized as a major source of uncertainty in the AIS’s response to climate change (*11*, *13*, *15*). Basal melt of Antarctic ice shelves is strongly influenced by the incursion of warm CDW, which occurs at ∼10-km spatial scales and daily to subdaily time scales (*47*) and cannot be faithfully simulated in a coarse resolution (∼100-km) ocean model typical of CMIP6 models. Recognizing these limitations, in this study, we use a simple parameterization scheme for basal melt rates, which assumes a quadratic dependence on the 400-m ocean temperature above the pressure melting point of ice (*T*_o_ − *T*_f_)

$$\mathrm{OM}=\mathrm{OMF}\left(\frac{K_{T}\rho_{w}C_{w}}{\rho_{i}L_{f}}\right)|T_{o}-T_{f}|(T_{o}-T_{f})$$

where ρ_w_ is the density of sea water, *C*_w_ is the specific heat capacity of sea water, ρ_i_ is the density of ice, *L*_f_ is the latent heat of fusion for ice, *T*_o_ is the ocean temperature at 400 m, *T*_f_ is the depth-dependent freezing point at the base of ice shelf, and *K*_T_ is a constant for ocean-ice turbulent heat transfer. OMF is a factor calibrated on the basis of observations, so that, under present-day climate, basal melt rate of Antarctic ice shelves modeled by the ISM totals to ∼1500 Gt year^−1^, falling within the observational range (*48*). This is admittedly a crude representation of ice shelf basal melting, which displays fine spatial patterns caused by subshelf ocean circulations. Fully resolving the complex processes involved in ice shelf basal melting is beyond the scope of this study.

For experiments with ISM parameters calibrated separately for each CMIP6 model, TPD and OMF are tuned so that, under modeled preindustrial climate conditions, the total surface/basal melting rate of Antarctic ice shelves both fit in the observational range (figs. S13 and S14). More detailed descriptions of the ISM are available in published work documenting the development of PSUICE3D (*5*, *18*).

### Marine ice cliff instability

The ISM’s ability to resolve migrating grounding lines naturally allows the onset of MISI (*49*), should climate perturbations drive the grounding line onto a reverse-sloping bed. Another mechanism that may lead to runaway ice sheet retreat, the MICI, involves mechanical failure of ice cliffs along terminating ice fronts where its subaerial height exceeds ∼80 m (*26*, *27*). Hydrofracturing and calving of shelf ice are proposed as potential processes that may expose tall ice cliffs prone to collapse. MICI-related processes including hydrofracturing of meltwater-covered ice shelves and mechanical failure of tall subaerial ice cliffs are parameterized in the ISM (*5*, *25*). Hydrofracturing is facilitated by deepening of the surface crevasses by meltwater and rainfall; ice cliff failure occurs where stresses at the ice front are diagnosed to exceed ice strength, assuming a linear ramp in calving rate (horizontal ice loss) up to a speed capped at 7.7 km year^−1^ at cliff heights reaching or exceeding 100 m. The maximum calving rate used here is the optimal value determined in large ensemble simulations constrained by modern (1992–2017, IMBIE) and paleo (LIG and Pliocene) contributions of the AIS to GMSL (*5*). In general, model physical parameters used in this study are the same as those documented in (*5*), except that the coefficient for ocean melt rate OMF and the melting point shift TPD in its PDD scheme are calibrated as specified above.

### Forcing the ISM with prescribed climate

Both observational climate data or output from CMIP6 climate models are used to provide atmospheric and oceanic boundary conditions for the ISM. The ISM uses three climate forcing fields: monthly near-surface air temperature (*T*_a_), monthly precipitation (*pr*), and ocean temperature at a depth of 400 m (*T*_o_). As sub-ice shelf cavities are not resolved in most CMIP6 models and ocean reanalysis datasets, for ISM grid cells with floating ice, 400-m ocean temperature of the nearest climate model grid cell is used for calculating ice shelf basal melt rates. Atmospheric fields from the relatively coarse CMIP6 model grid (nominal 1° latitude-longitude, ∼ 100 km) are bilinearly interpolated to the finer ISM grid (20 km), and a simple lapse-rate correction is applied to surface air temperature and precipitation, accounting for the difference between surface elevation prescribed in the climate model and that modeled in the ISM (*18*). Specifically, surface air temperature is shifted by Δ*T* = γΔ*z*, where γ = −0.008 Km^−1^ is the temperature lapse rate and precipitation is multiplied by a factor of 2^Δ*T*/10^

### Observational and CMIP6 climate forcing

Present-day climatological atmospheric forcing fields are derived from ERA5 (*22*), the latest generation atmospheric reanalysis data of the global climate from the European Centre for Medium-Range Weather Forecasts. Thirty-year mean (1981–2010) monthly near-surface air temperature and precipitation are used to force the ISM, as well as surface elevation in ERA5 for implementing lapse rate corrections for surface air temperature and precipitation. Annual mean ocean temperature at a 400-m depth, derived from World Ocean Atlas 2018 (WOA18) (*50*) 1981–2010 30-year mean, is fed to the ISM to calculate basal melt rates under ice shelves. Here, we treat reanalysis data as “observations,” because station-based meteorological data for Antarctica are still too sparse to provide whole-continent atmospheric boundary conditions for the ISM. Alternatives to ERA5 include other reanalysis datasets such as the Modern-Era Retrospective analysis for Research and Applications version 2 (MERRA2) and regional climate models such as the Regional Atmospheric Climate Model 2 (RACMO2) (*51*). Difference in near-surface air temperature between these datasets are most pronounced over the East Antarctic plateau and in winter, while summer temperature over the ice shelves shows much better consistency (figs. S1 to S3). This suggests that modeled processes related to summer ice surface melt, including hydrofracturing and collapse of ice shelves, are likely to have a weak dependence on the choice of dataset.

We select 36 models participating the CMIP6, which is overseen by the WCRP Working Group on Coupled Modeling. The selection is solely based on the availability of required variables (monthly near-surface air temperature, precipitation, surface elevation, and 400-m ocean temperature) for both historical and SSP5-8.5 scenarios as of October 2020. Although climate output from CMIP6 models are presented in various spatial resolutions, they are regridded onto the ISM grid before simulations. The ocean components in CMIP6 models usually do not resolve ice shelf cavities, so ocean temperatures under ice shelves are essentially missing from these climate models. In this study, water temperatures in contact with ice shelves are based on ocean temperature at the nearest ocean grid cell at the 400-m depth, which is around the core depth of the CDW.

### Single-ISM experiments

Experiment sets with the single-ISM setup are listed as Experiment nos. 0 to 7 in Table 1. Before conducting simulations forced by each CMIP6 model output, a 100,000-year inverse run under present-day observational climate (Exp. OBS_INV) was carried out to determine BSCs of the Antarctic bedrock (*25*). Specifically, BSCs are adjusted iteratively until the simulated ice thickness converges to that observed, while the grounding lines and ice shelves are fixed at their observed states. Updating the BSCs allows the ISM to simulate a more realistic geometry for the AIS in the control simulation under present-day climate.

To evaluate the effect of biases in modeled climate in the equilibrium AIS state, we first conduct a set of experiments (Exp. CMIP6_RAW_PI_CTL) where preindustrial climates are used as inputs to drive the ISM for 10,000 years so that it reaches quasi-equilibrium. In contrast, experiment suite CMIP6_BC_PI_CTL is designed to assess the spread in simulated AIS due to differences in bias-corrected CMIP6 preindustrial climates. Each experiment is forced with bias-corrected preindustiral climate from the respective CMIP6 model, with the first 10,000 years driven by the 1850–1869 20-year mean climatology and for the 5000 years thereafter driven by yearly recurrent 1850–1869 climate fields.

Exp. CMIP6_BC_1850-2100 is designed to assess the uncertainty in the AIS’s change and its contribution to GMSL since 1850, arising from CMIP6 models’ different climate responses to natural and anthropogenic forcing agents. CMIP6 climates are bias-corrected against present-day observations. Specifically, temperatures (near-surface air temperature and 400-m ocean temperature) are simply adjusted on the basis of their deviation ($\text{Δ}T={\bar{T}}^{\mathrm{PD}}-{\bar{T_{\mathrm{OBS}}}}^{\mathrm{PD}}$) from observations over the present-day (PD) period 1981–2010: *T*_BC_(*t*) = *T*(*t*) − Δ*T*. Existing research has demonstrated that methods for imposing precipitation anomalies, e.g., as fractional change from the reference state or as the change in absolute values, have profound consequences for the AIS’s projected mass balance (*52*). Here, modeled precipitation is adjusted by the ratio between the mean modeled annual precipitation over the period 1981–2010 (${\bar{P}}^{\mathrm{PD}}$) and the same quantity from observations (${\bar{P_{\mathrm{OBS}}}}^{\mathrm{PD}}$): $P_{\mathrm{BC}}(t)=P(t)\times {\bar{P_{\mathrm{OBS}}}}^{\mathrm{PD}}/{\bar{P}}^{\mathrm{PD}}$. A total of 250 years of model output from combined historical and SSP5-8.5 scenarios are bias-corrected and supplied yearly to the ISM to drive the evolution of the AIS from 1851 to 2100. With this approach, we have essentially removed the bias in each model’s present-day climate, focusing on the simulated past and future climate change.

Instead of using multimodel mean climate forcing (*13*), the ISM is forced with each CMIP6 model’s climate year by year, which preserves interannual variability that can be important for the ice sheet’s mass balance (*53*, *54*). Interannual variability in near-surface air temperature is especially crucial for the ice sheet’s SMB and the stability of ice shelves due to its role in surface melting and meltwater production, which could potentially trigger hydrofracturing.

Exp. CMIP6_BC_1850-2100_NO_MICI uses the same setup as Exp. CMIP6_BC_1850-2100, except that MICI-related processes are turned off in the ISM, which provides a reference case for assessing the effect of MICI on future Antarctic ice loss following the high-emission SSP5-8.5 scenario. Exp. CMIP6_BC_MMM and Exp. CMIP6_BC_MMM+OBS are similarly forced with multimodel mean (MMM) bias-corrected CMIP6 climate, except that the latter is forced by observational climate data during the period 1980–2019. They are designed for assessing the effect of observed multidecadal trends in Antarctic climate—especially the warming trend for the CDW—on transient changes in the AIS.

Designed for ice sheet modeling forced “offline” by climate model output, the framework used here precludes important ice sheet–climate feedbacks, including the ice-albedo feedback and ice-ocean interactions associated with fresh water discharged from Antarctica into the Southern Ocean. These feedbacks are potentially important to both the trajectory of warming over Antarctica and the projected change in AIS and GMSL (*41*). However, as far as this century is concerned, ice-albedo feedback is dominated by the changes in sea ice extent and snow cover, which are explicitly simulated by climate models, and the changes in the extent of ice sheet and ice shelves are not yet large enough to have a notable impact on the radiative budget. Antarctic freshwater discharge is projected to steadily increase over this century, affecting upper ocean stratification and sea ice formation. Ice sheet fresh water may be associated with both positive and negative feedbacks (*42*, *43*, *55*) but is difficult to account for in our modeling framework. Proper inclusion of these feedbacks can only be achieved in fully coupled ice sheet–climate models and the ISM, which would be impractical for this study considering the large number of climate models used here.

### Experiments with ISM parameters separately calibrated for each CMIP6 model

This series of experiments are designed to assess the effect of ISM parameter calibration on the projected Antarctic ice loss. Specifically, parameter TPD in the ISM’s PDD scheme is tuned so that, under each CMIP6 model’s present-day climate (averaged over the period 1980–2100), the modeled total rate of meltwater production on Antarctic ice shelves is 100 Gt year^−1^, within the observational range (Exp. CMIP6_RAW_TPD) (*46*); the factor governing oceanic melt rates (OMF) is tuned so that the calculated Antarctic total sub-ice melt is around 1500 Gt year^−1^, falling within the envelope estimated on the basis of observational data (Exp. CMIP6_RAW_OMF) (*48*). With observation-constrained parameters TPD and OMF (figs. S13 and S14), we put the ISM on an 80,000-year inverse run under respective CMIP6 model’s preindustrial climate, during which the BSC is adjusted periodically so that the simulated AIS gradually converges to its present-day state (Exp. CMIP6_RAW_INV). This approach essentially results in 36 different ISMs, each tuned so that the ISM reproduces observed Antarctic surface and sub-ice melt rates under respective CMIP6 model’s present-day climate and simulates a realistic AIS similar to that observed under the CMIP6 model’s preindustrial climate.

Tuning the ISM for each CMIP6 model allows the ISM to run under raw climate model output and simulates a realistic AIS (fig. S15). Inverse runs in this framework, however, performs not as well as Exp. OBS_INV for some CMIP6 climates (fig. S15), resulting in apparent differences in ice sheet geometries. In this framework, to assess the changes in the AIS due to transient changes in modeled climate, we run two sets of experiments: Both start from a present-day ice sheet configuration and run for 250 years, but one (Exp. CMIP6_RAW_PI_CTL2) is forced with raw preindustrial climate (recurrent 1850–1869 transient climate fields) and the other (Exp. CMIP6_RAW_1850-2100) with yearly 1850–2100 raw climate fields. Subtracting drifts identified in CMIP6_RAW_PI_CTL2 from output in respective CMIP6_RAW_1850-2100 runs, we can isolate changes in the AIS due to the evolving climate in each CMIP6 model.

## Supplementary Materials

### This PDF file includes:

Sections S1 to S6 Figs. S1 to S16
