# Supplementary material for: Climate model differences contribute deep uncertainty in future Antarctic ice loss
Source: Sci Adv. 2023 Feb 15;9(7):eadd7082. doi: 10.1126/sciadv.add7082 (PMC9931235; doi:10.1126/sciadv.add7082)
Supplement: Supplementary file 1 — Sections S1 to S6 Figs. S1 to S16 [file sciadv.add7082_sm.pdf]

Science Advances
AAAS

Supplementary Materials for

Climate model differences contribute deep uncertainty in future Antarctic ice loss

Dawei Li *et al.*

Corresponding author: Dawei Li, lidavvei@sjtu.edu.cn

Sci. Adv. **9**, eadd7082 (2023)
DOI: 10.1126/sciadv.add7082

This PDF file includes:

Sections S1 to S6
Figs. S1 to S16

1 Comparison of monthly surface air temperature from reanalysis datasets

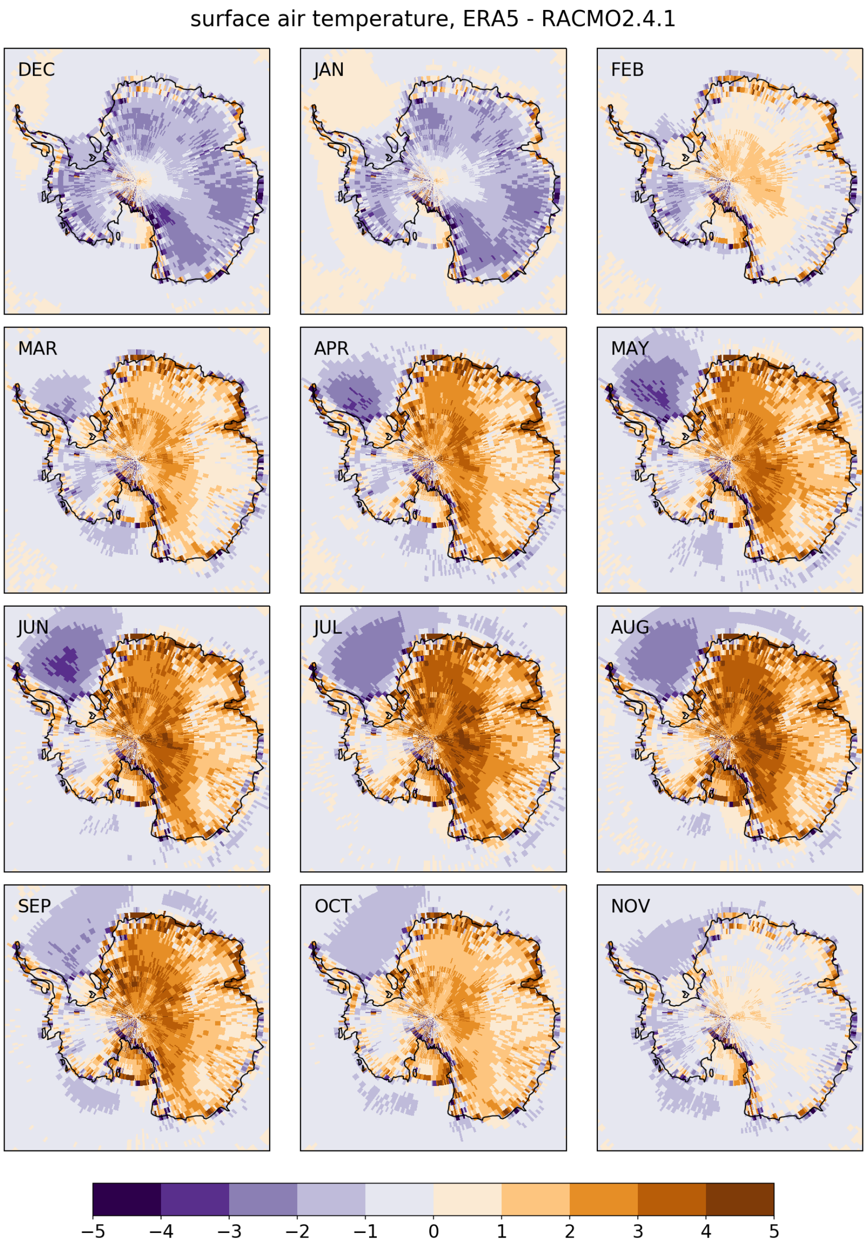

Fig S. 1: Difference in monthly surface air temperature (°C) between ERA5 and RACMO2.4.1.

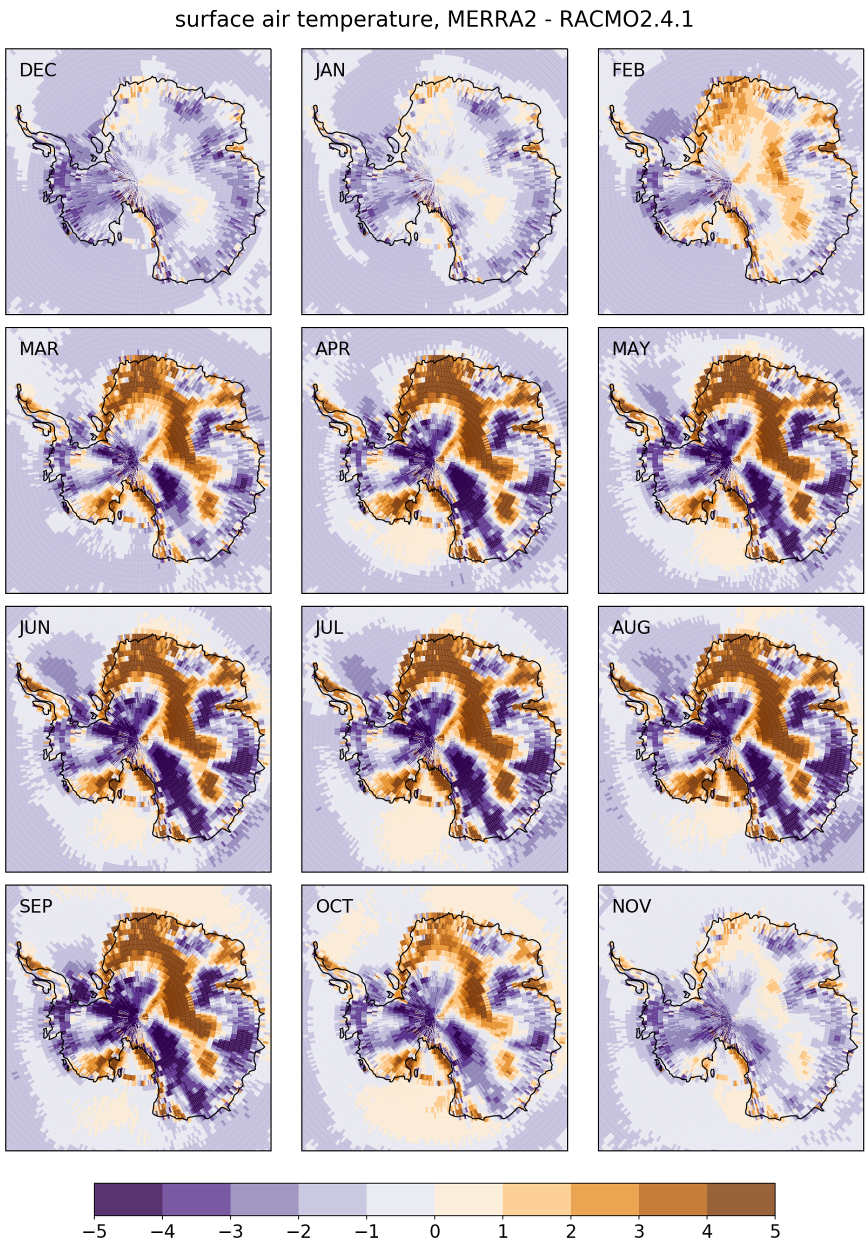

Fig S. 2: Difference in monthly surface air temperature (°C) between MERRA-2 and RACMO2.4.1.

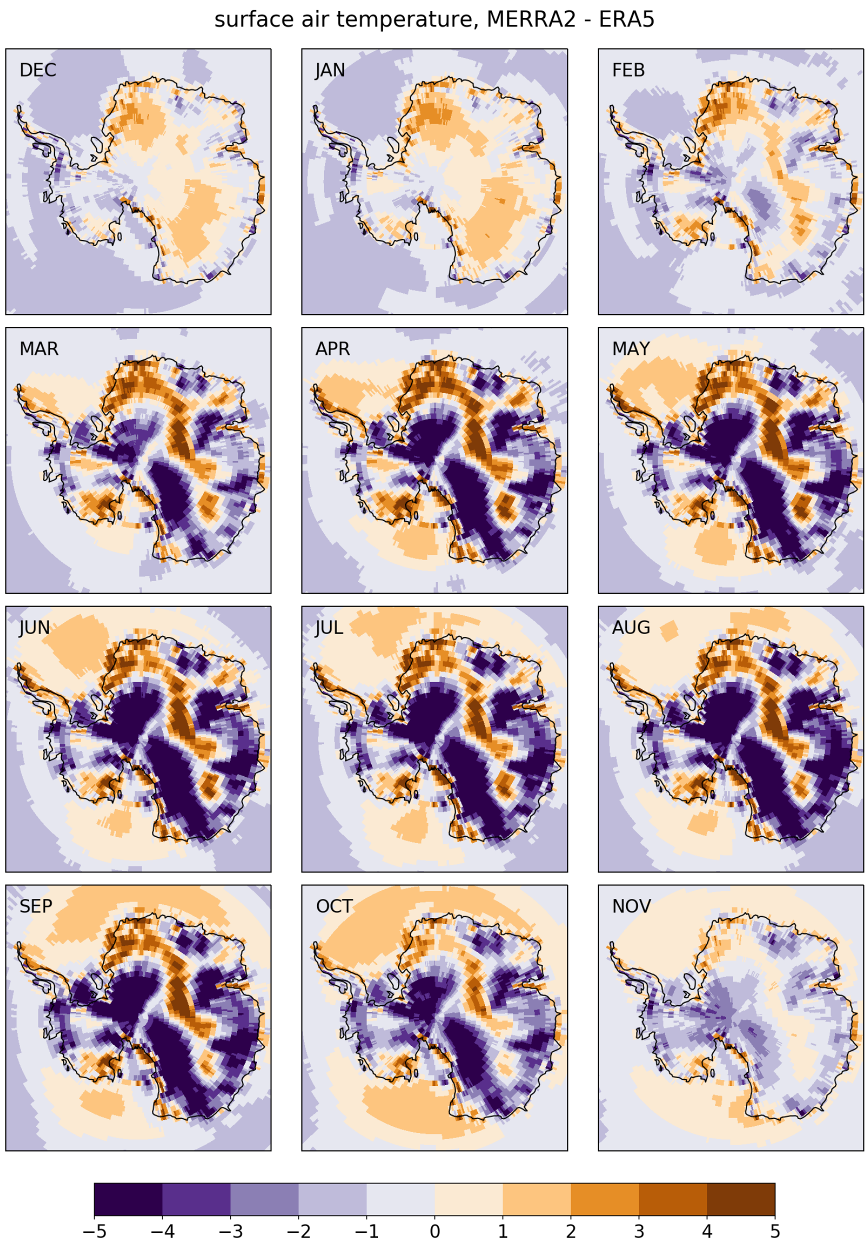

Fig S. 3: Difference in monthly surface air temperature (°C) between MERRA-2 and ERA5.

2 Comparison of climatological fields from CMIP6 models

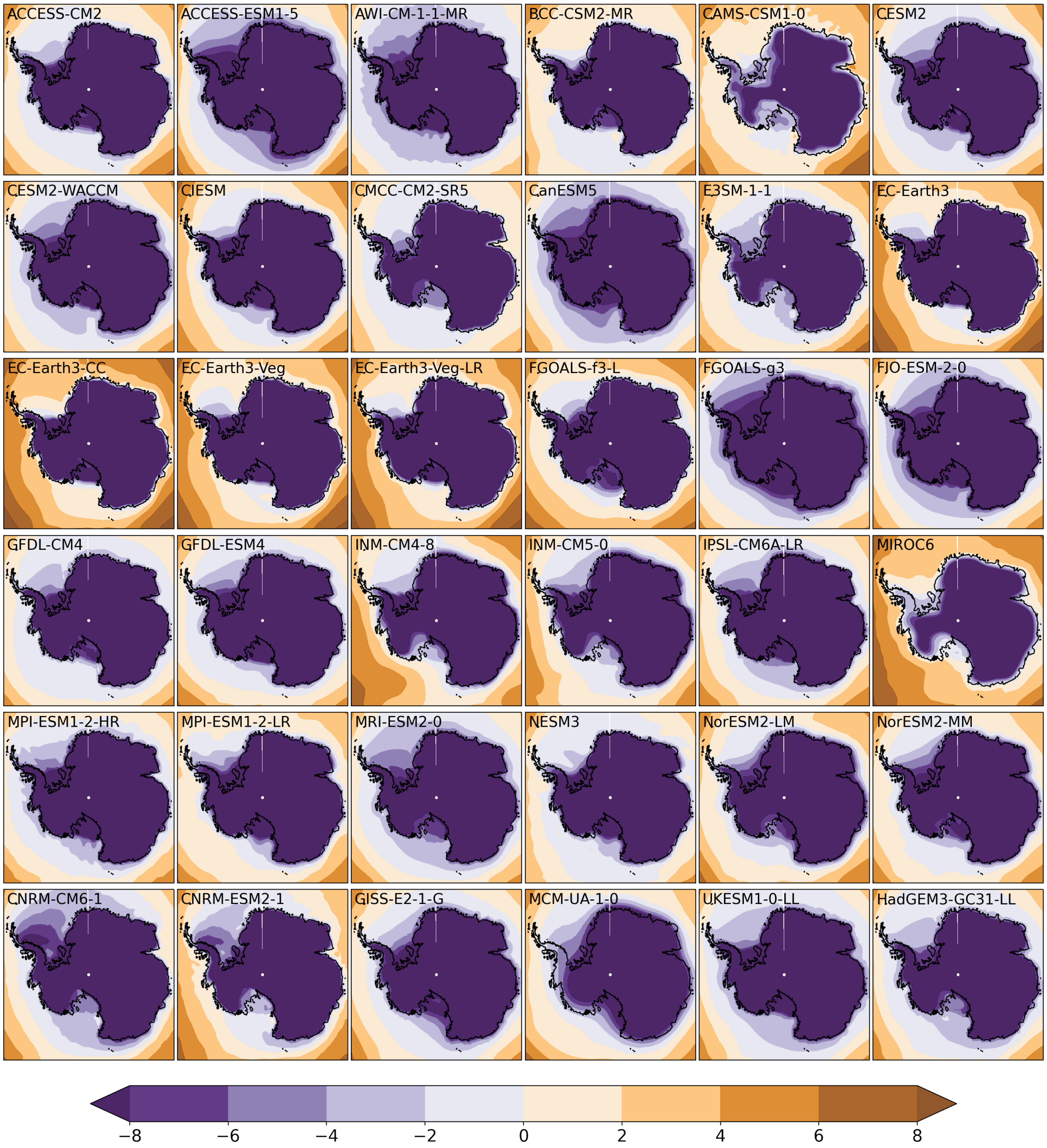

Fig S. 4: DJF near-surface air temperature (°C) from 36 CMIP6 models averaged over the *historical* period 1981-2010.

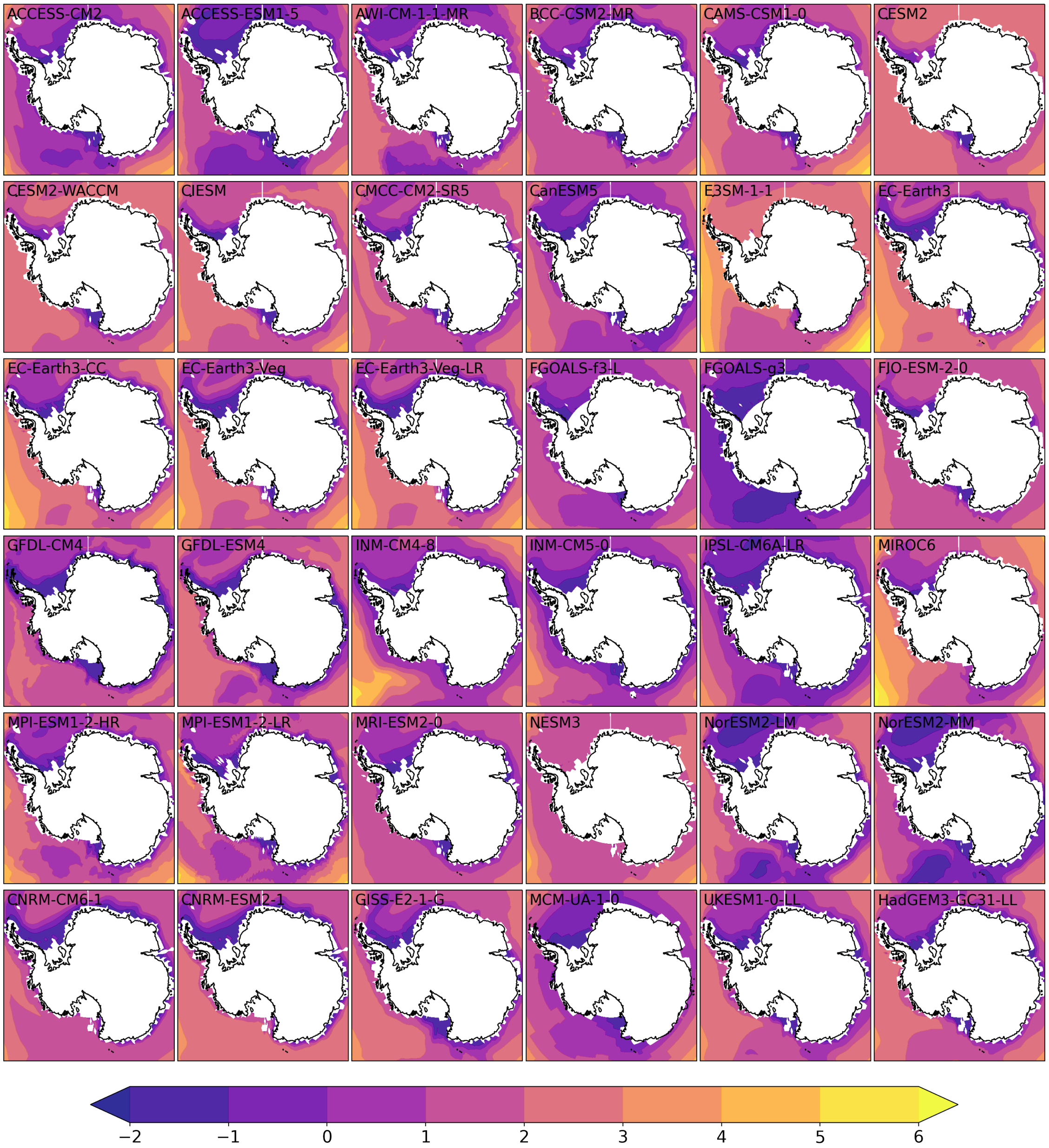

Fig S. 5: Annual mean 400m ocean potential temperature (°C) from 36 CMIP6 models averaged over the *historical* period 1981-2010.

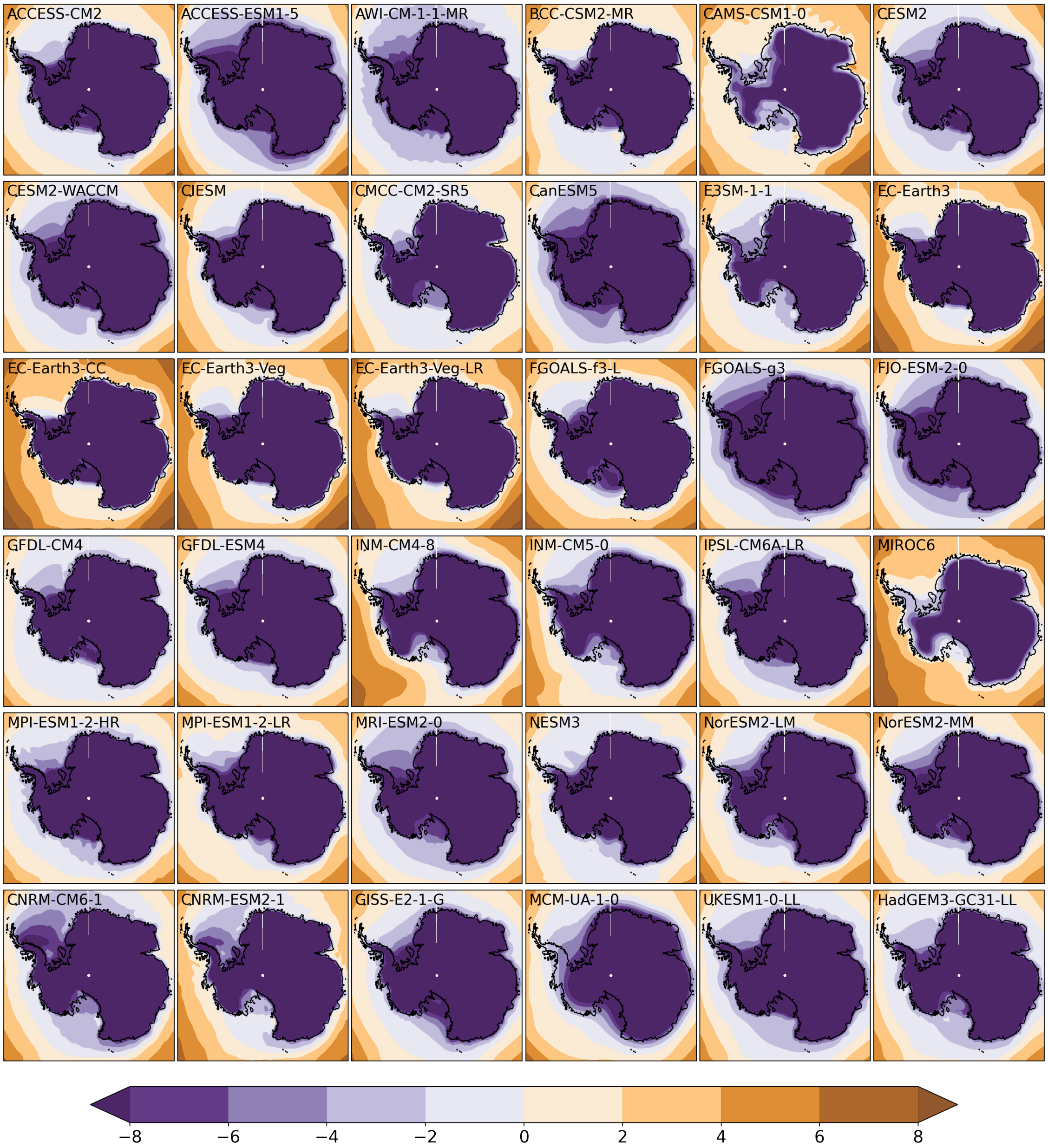

Fig S. 6: Annual-mean precipitation (m yr^{-1}) from 36 CMIP6 models averaged over the *historical* period 1981-2010.

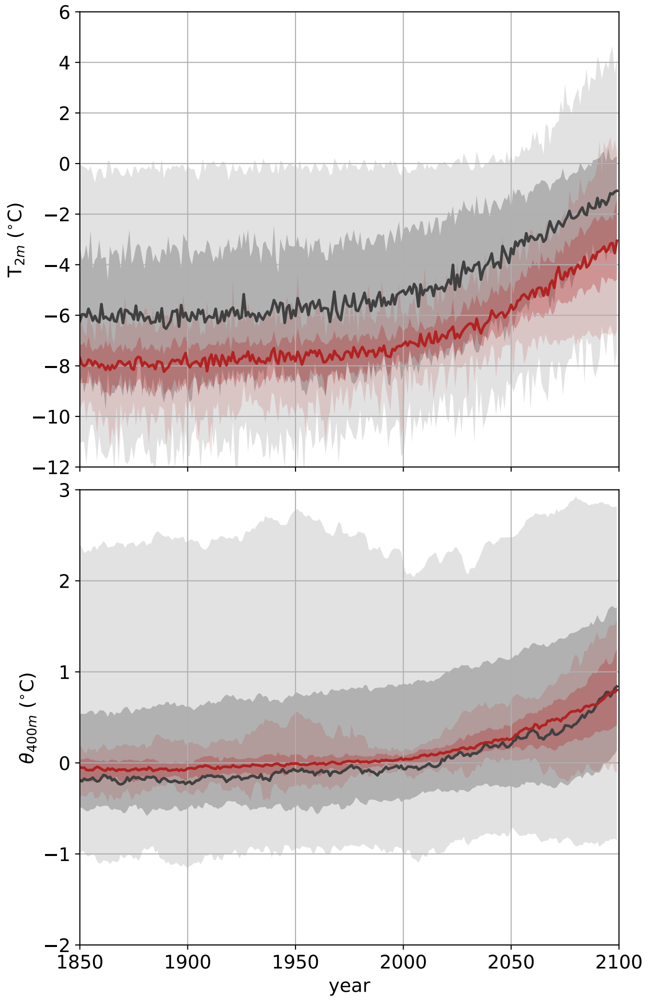

Fig S. 7: Change in near-surface air temperature (top) and 400m ocean potential temperature (bottom) from 36 CMIP6 models in combined *historical + ssp5-8.5* scenarios. averaged over the *historical* period 1981-2010. Shades of gray represent the original model output, and shades of red represent bias-corrected (against 1981-2010 observational data) time series. In each panel, the full spread (0 percentile to 100 percentile) in 36 models is shaded in light colors, 16 to 84 percentile in darker colors, and the median in solid lines.

3 Effects of MICI-related processes on the response of the AIS to climate warming

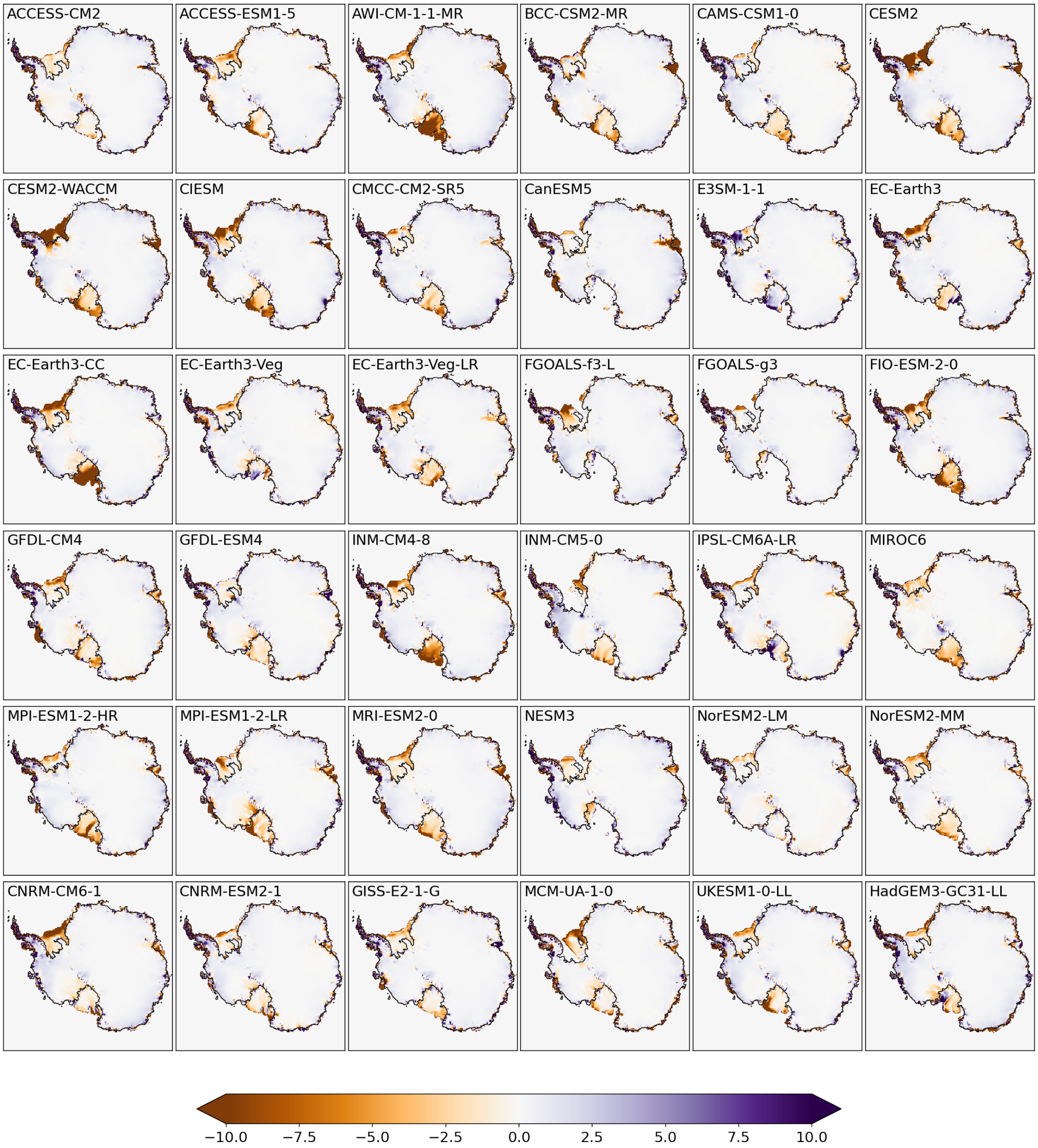

Fig S. 8: Change in ice thickness since 1850 by year 2020 in simulations without *hydrofracturing* or *ice cliff failure* and transiently forced by bias-corrected historical+ssp585 climate fields from 36 CMIP6 models. These are essentially the same as Fig. 3a but with MICI-related processes turned off.

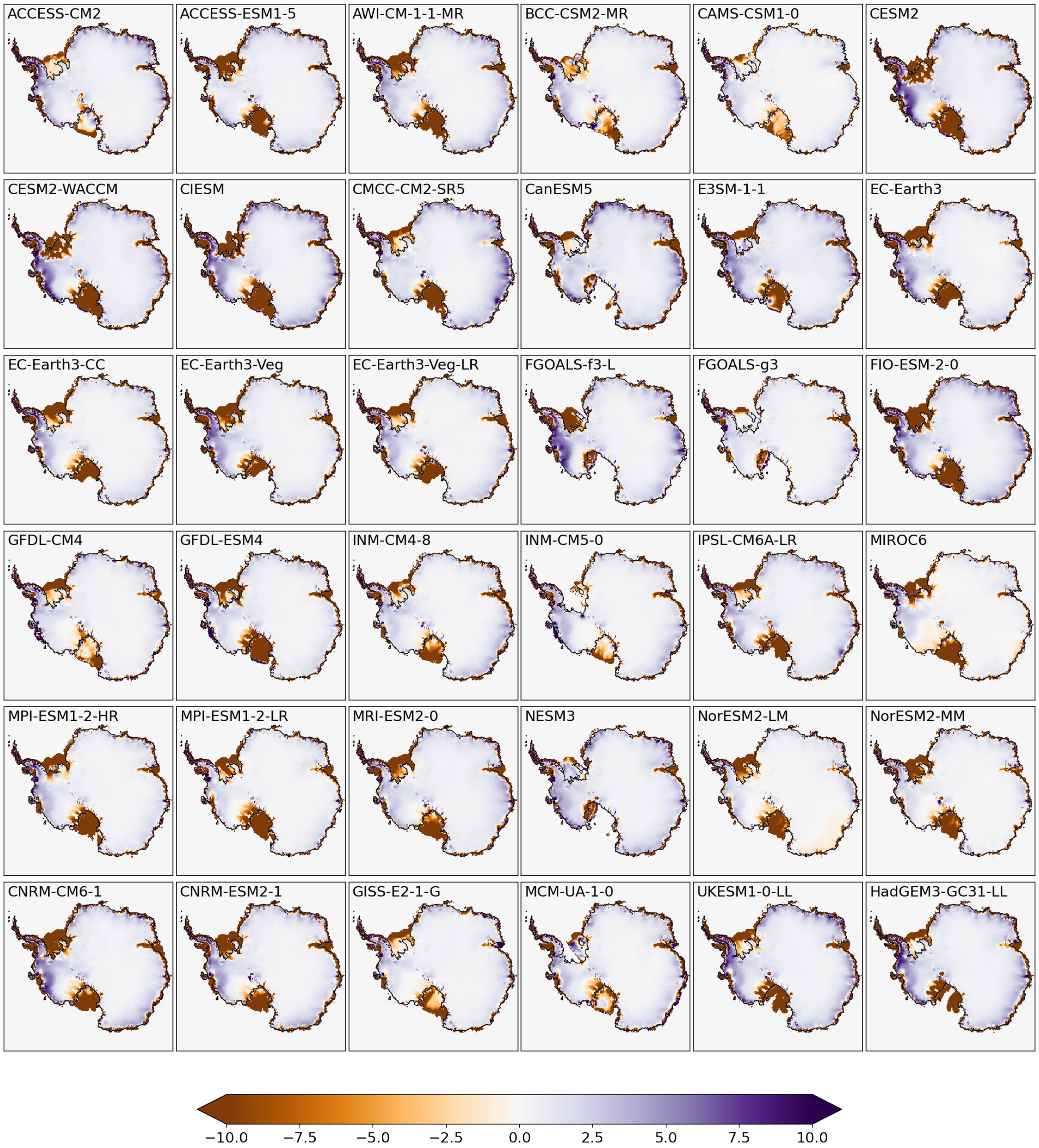

Fig S. 9: Change in ice thickness since 1850 by year 2100 in simulations without *hydrofracturing* or *ice cliff failure* and transiently forced by bias-corrected historical+ssp585 climate fields from 36 CMIP6 models. These are essentially the same as Fig. 3b but with MICI-related processes turned off.

4 Preindustrial control ISM simulations forced by CMIP6 models

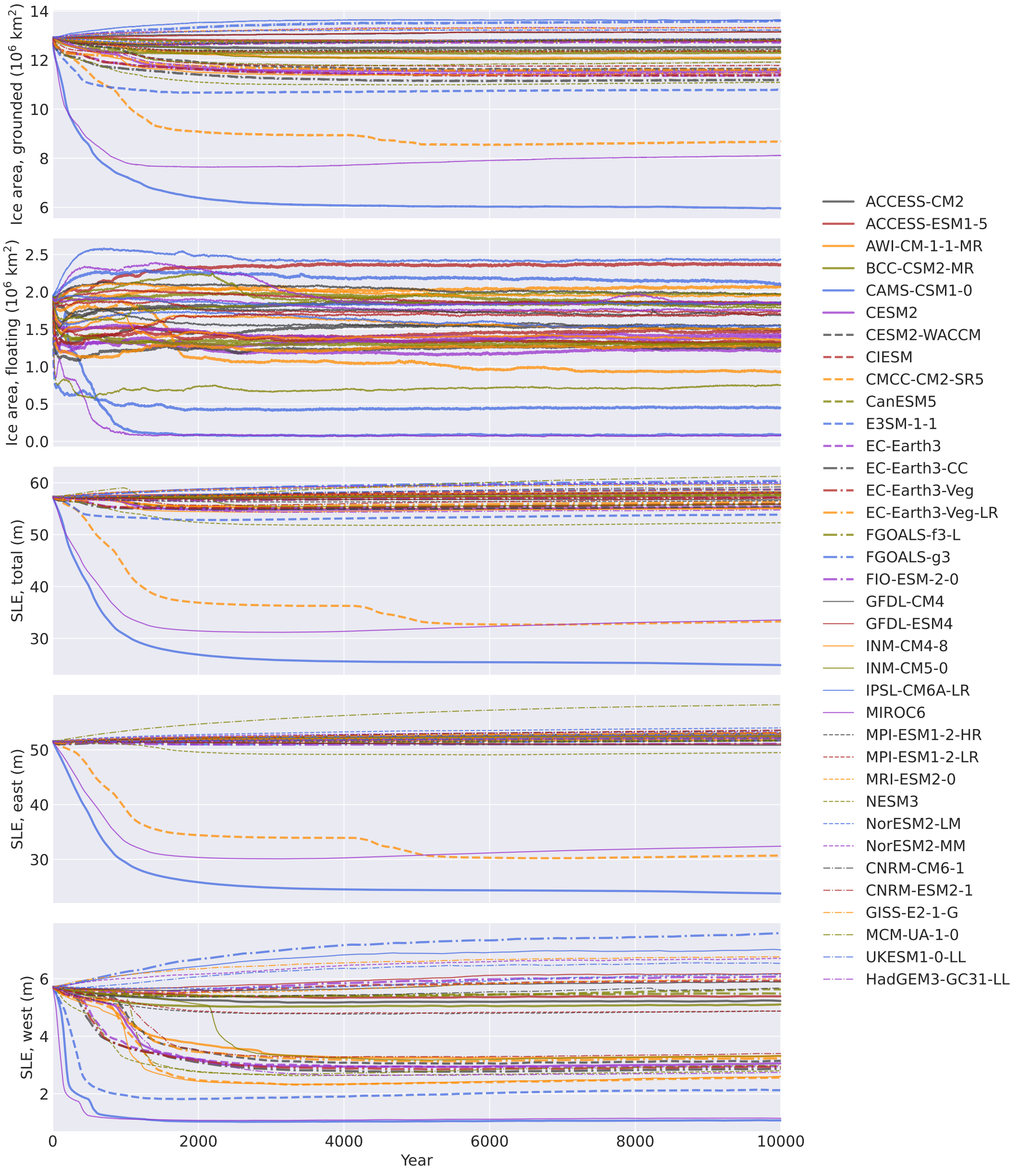

Fig S. 10: Time series of selected variables for the AIS in ISM control simulations forced by original climatological fields from 36 CMIP6 models.

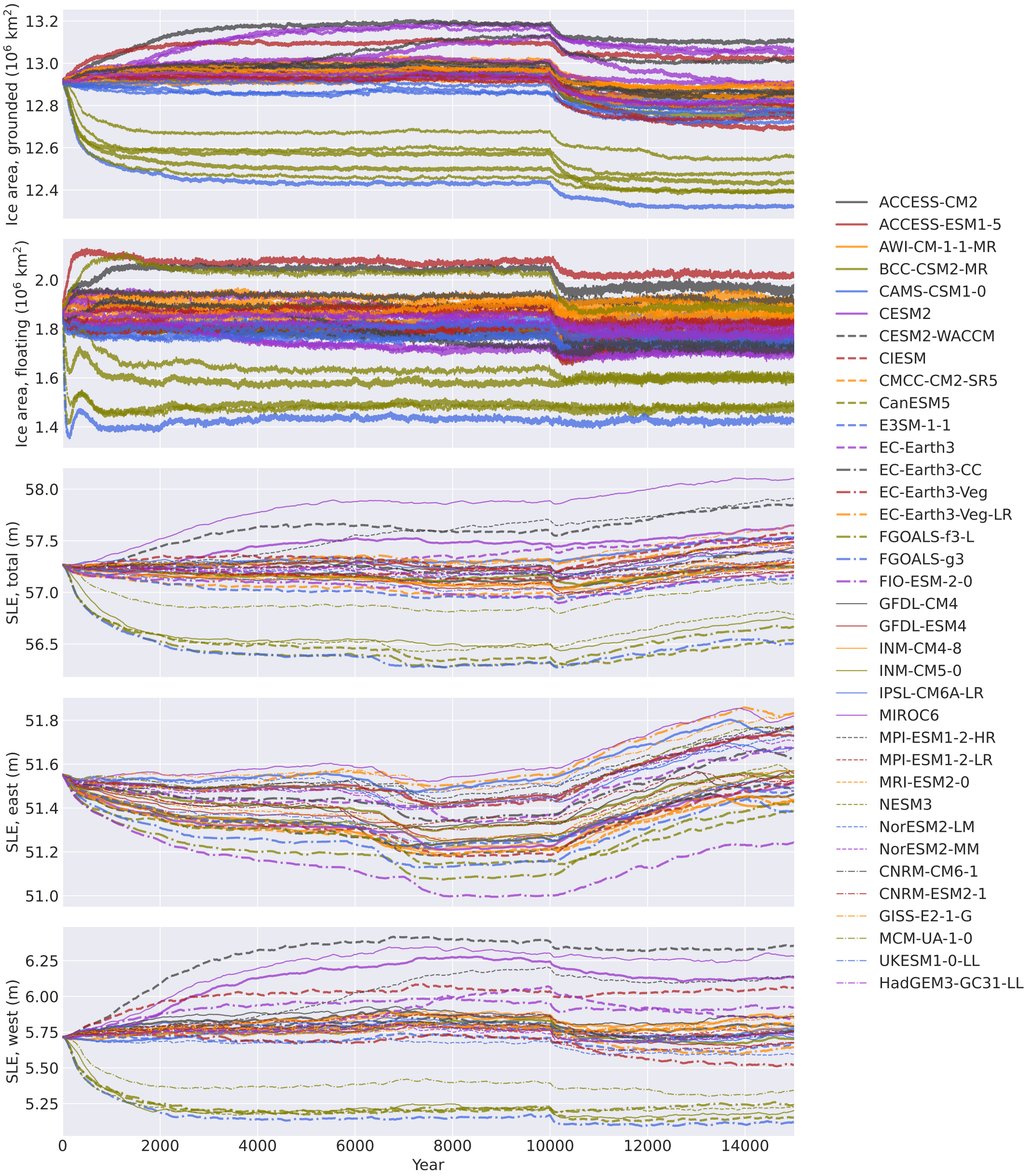

Fig S. 11: Time series of selected variables for the AIS in ISM control simulations forced by bias-corrected (against 1981-2010 observational data) climates from 36 CMIP6 models. The ISM is forced by 1850-1869 bias-corrected climatologies during the first 10,000 years, and by recurrent 1850-1869 yearly climates thereafter for 5000 years.

5 Transient ISM simulations forced by CMIP6 multi-model mean and observed climate fields

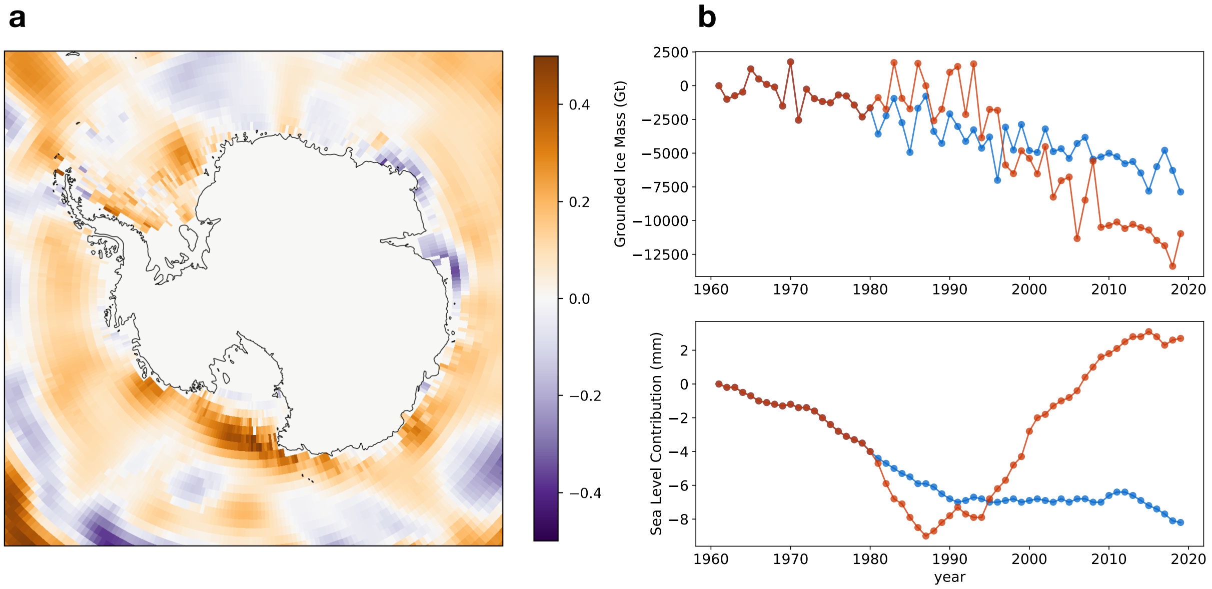

Fig S. 12: Left: Difference in 400-m ocean temperature between the periods 1985-1994 and 2005-2017 from WOA18; Right: Time series of grounded ice mass (upper) and Antarctic contribution to GMSL (lower) in transient ISM simulations forced by CMIP6 multi-model mean (red lines) and observed climate fields (blue lines) from ERA5 and WOA18.

6 Simulations forced by raw CMIP6 climate fields with the ISM calibrated respectively for each model

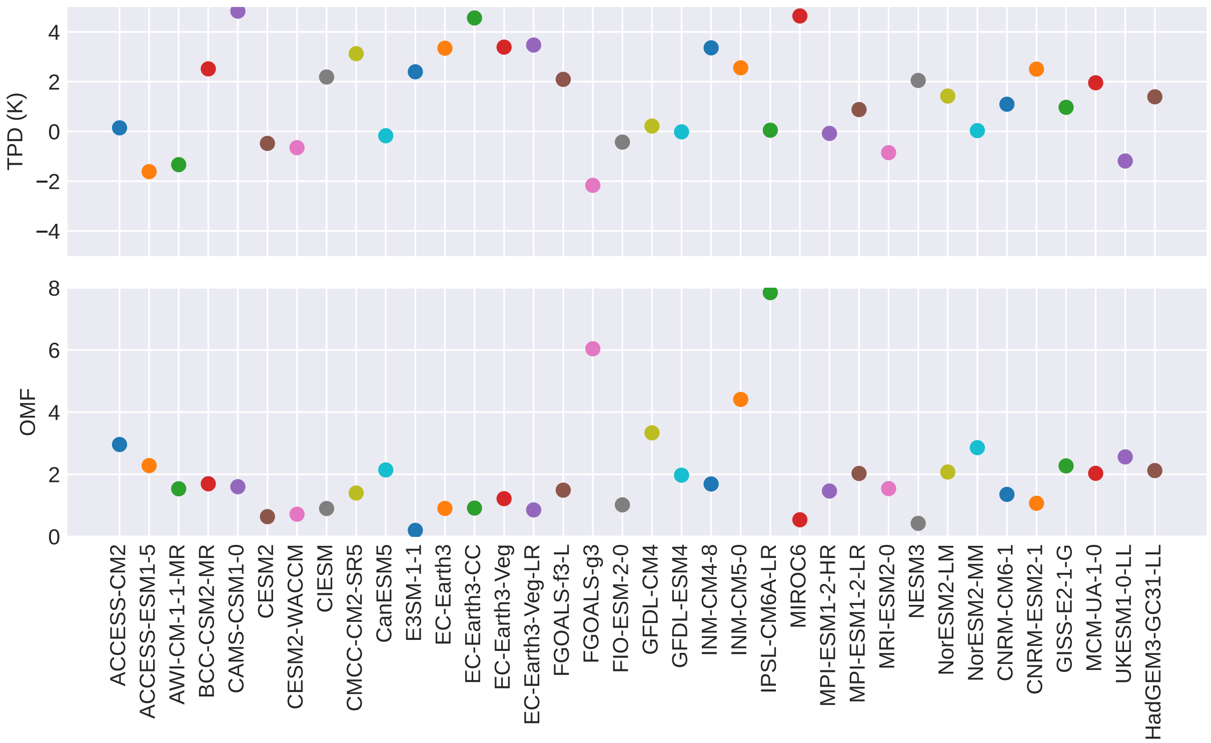

Fig S. 13: Parameters tuned for each CMIP6 model. Upper panel shows $\mathcal{TPD}$, a parameter in the ISM's surface ablation scheme (a Positive Degree Day model) that shifts the melting point of ice and snow; Bottom panel shows $\mathcal{OMF}$, the coefficient governing the melting rate at the base of ice shelves.

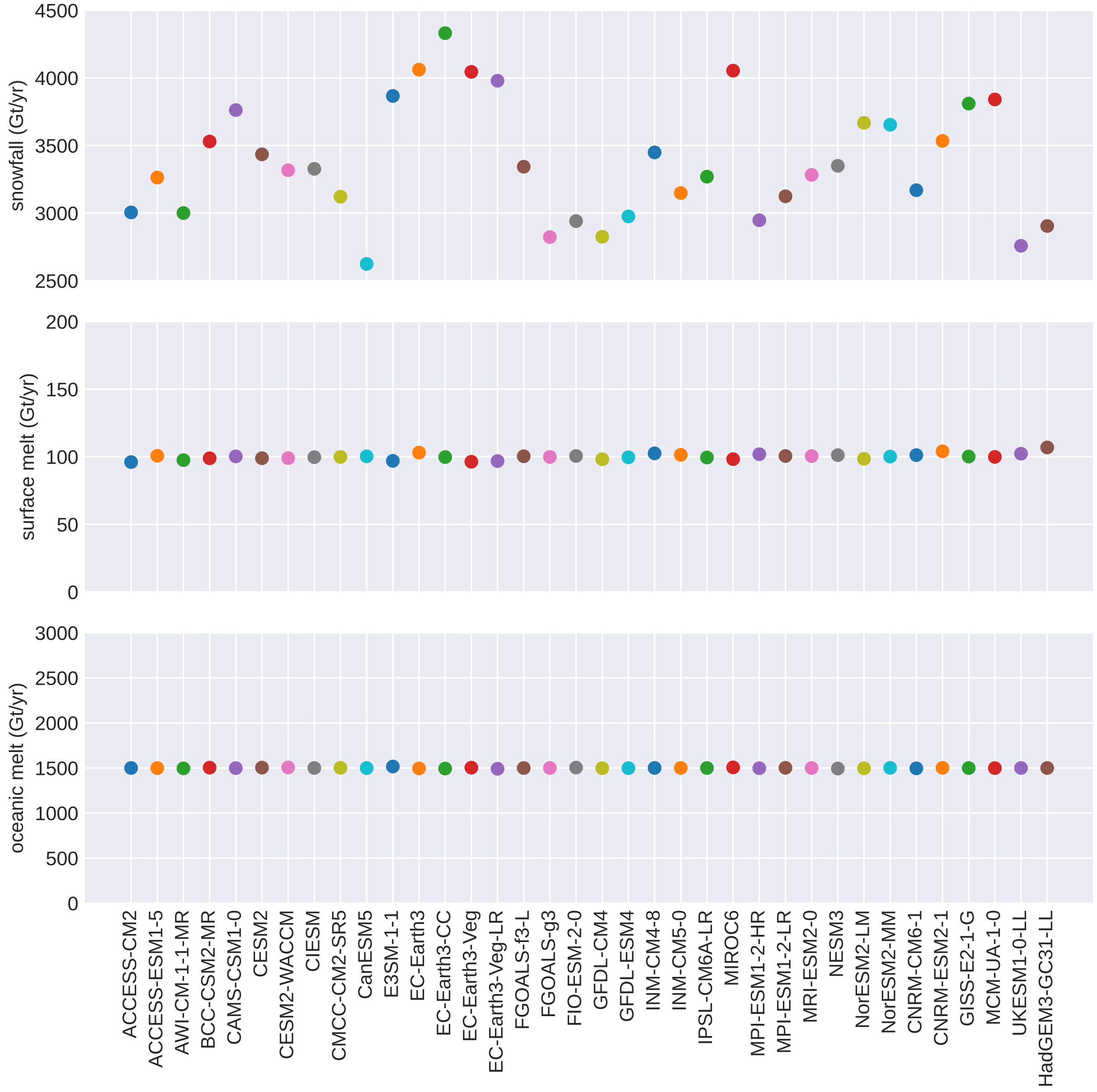

Fig S. 14: Rates of annual snow accumulation over the AIS (top), meltwater production on Antarctic ice shelves (middle), and ice shelf basal melt (bottom) in the first year of ISM runs under each CMIP6 model's present-day climate (1981-2010 historical scenario). Tuning targets for surface meltwater production and basal melt are 100 Gt/yr and 1500 Gt/yr, respectively.

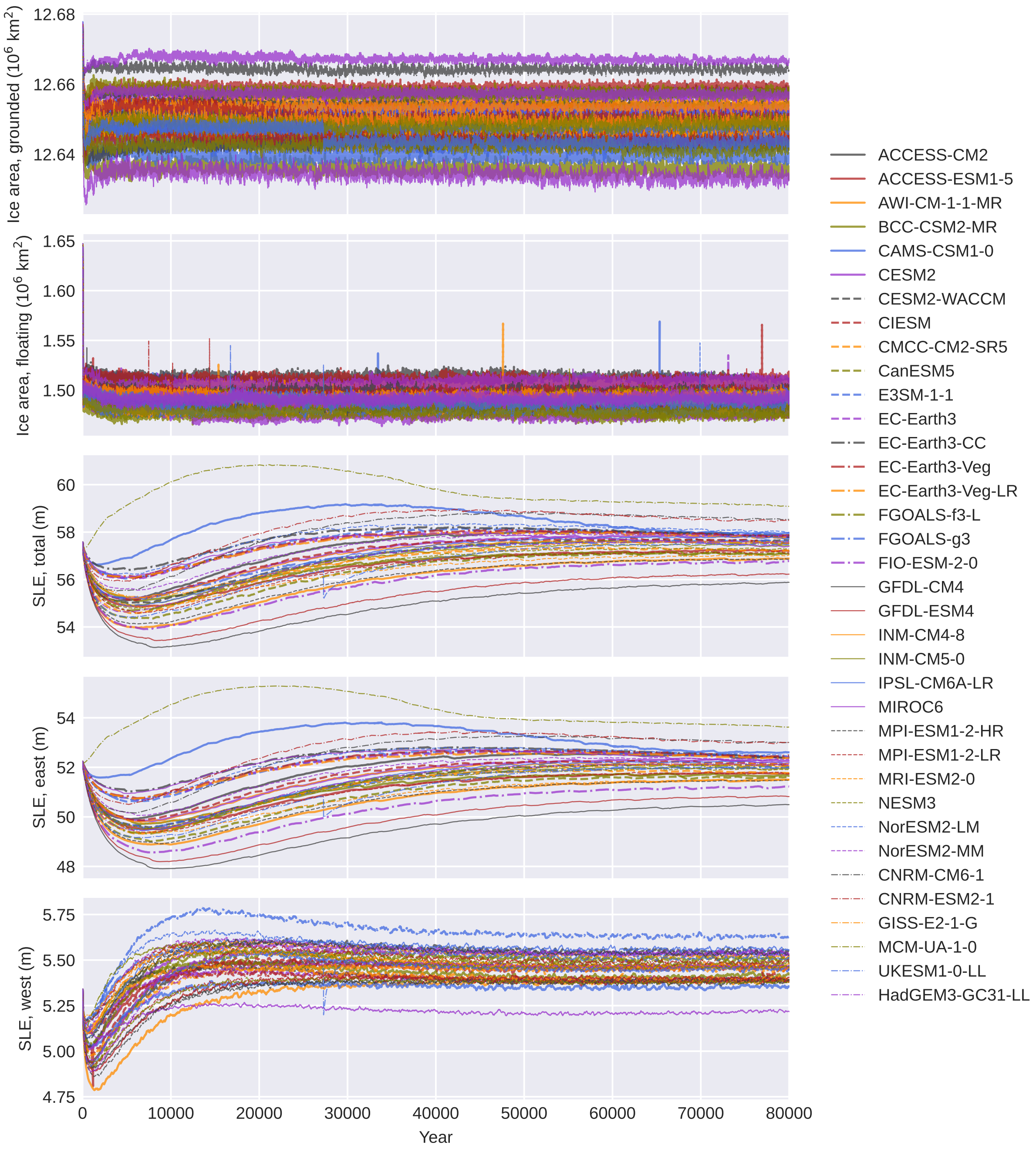

Fig S. 15: Time series of ice area (grounded and floating) and the AIS's SLE (total, EAIS, and WAIS) in inverse runs for each CMIP6 climate model. Basal slide coefficients are adjusted periodically at each grid point based on the trend in local ice thickness.

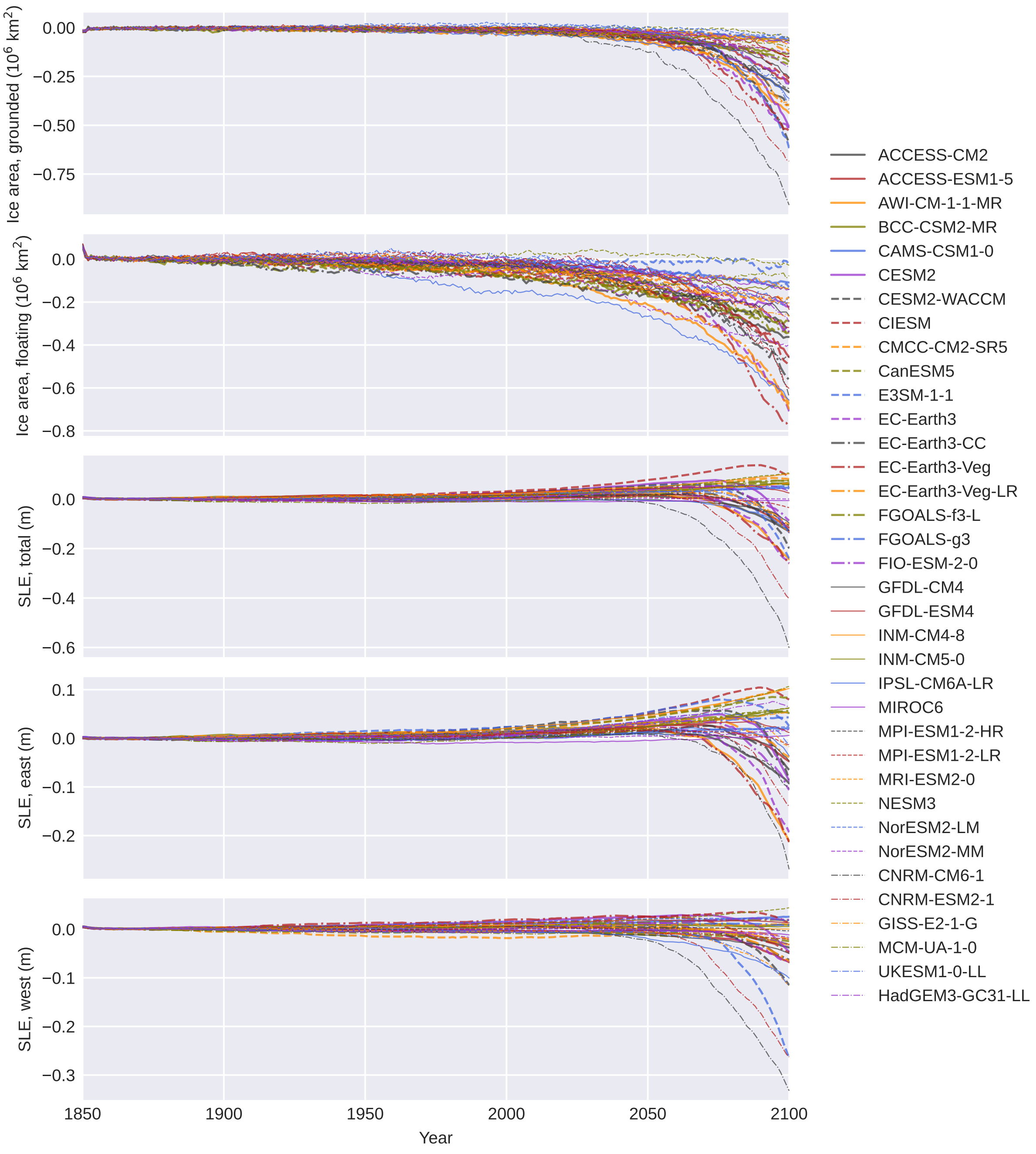

Fig S. 16: Time series of ice area (grounded and floating) and the AIS's SLE (total, EAIS, and WAIS) in transient runs forced by raw yearly climate fields from each CMIP6 model. This set of runs are essentially the same as CMIP6_BC_1850-2100 except that the ISM is tuned respectively for each CMIP6 model, and raw climate fields are used to drive the ISM.
